# Phylogeography of *Bellamya* (Mollusca: Gastropoda: Viviparidae) snails on different continents: contrasting patterns of diversification in China and East Africa

**DOI:** 10.1186/s12862-019-1397-0

**Published:** 2019-03-21

**Authors:** Qian H. Gu, Martin Husemann, Hui H. Wu, Jing Dong, Chuan J. Zhou, Xian F. Wang, Yun N. Gao, Man Zhang, Guo R. Zhu, Guo X. Nie

**Affiliations:** 1 State Key Laboratory of Developmental Biology of Freshwater Fish, College of Life Sciences, Hunan Normal University, No. 36 Lushan Road, Changsha City, 410081 Hunan People’s Republic of China; 2 College of Fisheries, Engineering Technology Research Center of Henan Province for Aquatic Animal Cultivation, Henan Normal University, Xinxiang, 453007 Henan People’s Republic of China; 3 Centrum für Naturkunde, Universität Hamburg, 20146 Hamburg, Germany

**Keywords:** *Bellamya*, ‘out of Asia’, Vicariance, Species radiation, Landscape dynamics

## Abstract

**Background:**

Species diversity is determined by both local environmental conditions that control differentiation and extinction and the outcome of large-scale processes that affect migration. The latter primarily comprises climatic change and dynamic landscape alteration. In the past few million years, both Southeast Asia and Eastern Africa experienced drastic climatic and geological oscillations: in Southeast Asia, especially in China, the Tibetan Plateau significantly rose up, and the flow of the Yangtze River was reversed. In East Africa, lakes and rivers experienced frequent range expansions and regressions due to the African mega-droughts. To test how such climatic and geological histories of both regions relate to their respective regional species and genetic diversity, a large scale comparative phylogeographic study is essential. *Bellamya*, a species rich freshwater snail genus that is widely distributed across China and East Africa, represents a suitable model system to address this question. We sequenced mitochondrial and nuclear DNA for members of the genus from China and used published sequences from Africa and some other locations in Asia to investigate their phylogeny and distribution of genetic diversity.

**Results:**

Our phylogenetic analysis revealed two monophyletic groups, one in China and one in East Africa. Within the Chinese group, *Bellamya* species show little genetic differentiation. In contrast, we observe fairly deep divergence among the East African lakes with almost every lake possessing its unique clade. Our results show that strong divergence does not necessarily depend on intrinsic characteristics of a species, but rather is related to the landscape dynamics of a region.

**Conclusion:**

Our phylogenetic results suggest that the *Bellamya* in China and East Africa are independent phylogenetic clades with different evolutionary trajectories. The different climate and geological histories likely contributed to the diverging evolutionary patterns. Repeated range expansions and regressions of lakes likely contributed to the great divergence of *Bellamya* in East Africa, while reversal of the river courses and intermingling of different lineages had an opposite effect on *Bellamya* diversification in China.

**Electronic supplementary material:**

The online version of this article (10.1186/s12862-019-1397-0) contains supplementary material, which is available to authorized users.

## Background

The roles that past climate fluctuations and geological events have played in species diversification and distribution are of main interest in phylo- and biogeography [1–3]. One major consequence of drastic climatic changes and geological activities are ecosystem and landscape reconfigurations. An example of a specifically large event is the drastic uplift of the Tibet Plateau and the resulting Asian monsoons, which largely re-shaped the landscape and affected the climate of eastern Asia [4–7]. These events are also considered an important driving force of vicariant speciation and intraspecific divergence in many freshwater species in the region [8–10], including salamanders and frogs [11–13]. Such rearrangements in the landscape can open up new migration corridors, facilitate the colonization of new habitats, and lead to novel species interactions [14]. The dynamic change of a landscape is considered one of the fundamental forces driving speciation [15, 16]; yet, species life history traits also have an impact on the speciation rate [17–19]. Strong philopatry, low dispersal ability and specific breeding strategies promote rapid divergence of local populations [20, 21], whereas high dispersal leads to admixture and prevents such differentiation [22, 23].

A first step to identify the drivers of diversification is to establish a robust phylogeny. This approach is especially powerful when closely related taxa are compared. Close relatives are at early stages of their evolution, and their differences may still reflect independent selectional forces that had operated upon them.

As one of the widely distributed freshwater gastropods in Asia, India and Africa, *Bellamya* has been identified as relatively recent radiation [24, 25]. This is an optimal situation in order to test for the potential drivers of speciation in multiple geographic regions with different patterns of paleo-climatic fluctuations and geological events. Several other characteristics make *Bellamya* a great system to study the drivers of speciation: firstly, as freshwater gastropods, the large numbers of well-preserved shells serve as excellent fossil calibration points for molecular dating [24]. Secondly, their dispersal abilities are limited, rendering the passing of aquatic or terrestrial barriers difficult [26]. Thirdly, the fossils discovered in Southeast Asia [27], Mid-East [28, 29] and East Africa [14], suggest that *Bellamya* was found in these regions before the Pleistocene, when the climate oscillations were most intense. They can passively migrate by drainage evolution [30]. These characteristics offer excellent opportunities to test phylogeographic hypotheses and provide a powerful system to disentangle the relative importance of various climatic and geographic factors potentially involved in species diversification [31].

In this study, we chose the genus *Bellamya*, as our model system, to test how climatic and geological events have influenced the genetic diversity of this taxon on a large geographic scale. *Bellamya* is a species-rich genus of freshwater snails occurring in Asia, India and Africa [24, 25]. Previous phylogenetic studies on the East African radiation have found that each of the great lakes possesses its own endemic species flock [24, 25]. The African and Asian species of *Bellamya* are sister groups with relatively low divergence contradicting an ancient Gondwanan vicariance [24]. However, little is known regarding the relationship within the Southeast Asian *Bellamya* lineage despite its diversitiy; at least 18 species have been reported from China [32].

Here we further investigate the relationships between Chinese and East African *Bellamya* lineages. Specifically, we intend to examine the patterns of diversification in Chinese and East African *Bellamya* lineages to test three hypotheses:

1. The Chinese and East African lineages represent monophyletic entities.
2. Similar to East Africa, Chinese *Bellamya* split into several distinct and isolated lineages.
3. Similar to other freshwater taxa, *Bellamya* originated in Africa and dispersed to Asia where it diverged from the African population due to strong geographic isolation.

## Results

### Geometrical morphology and ultrastructure of radulae

**Fig. 1:**
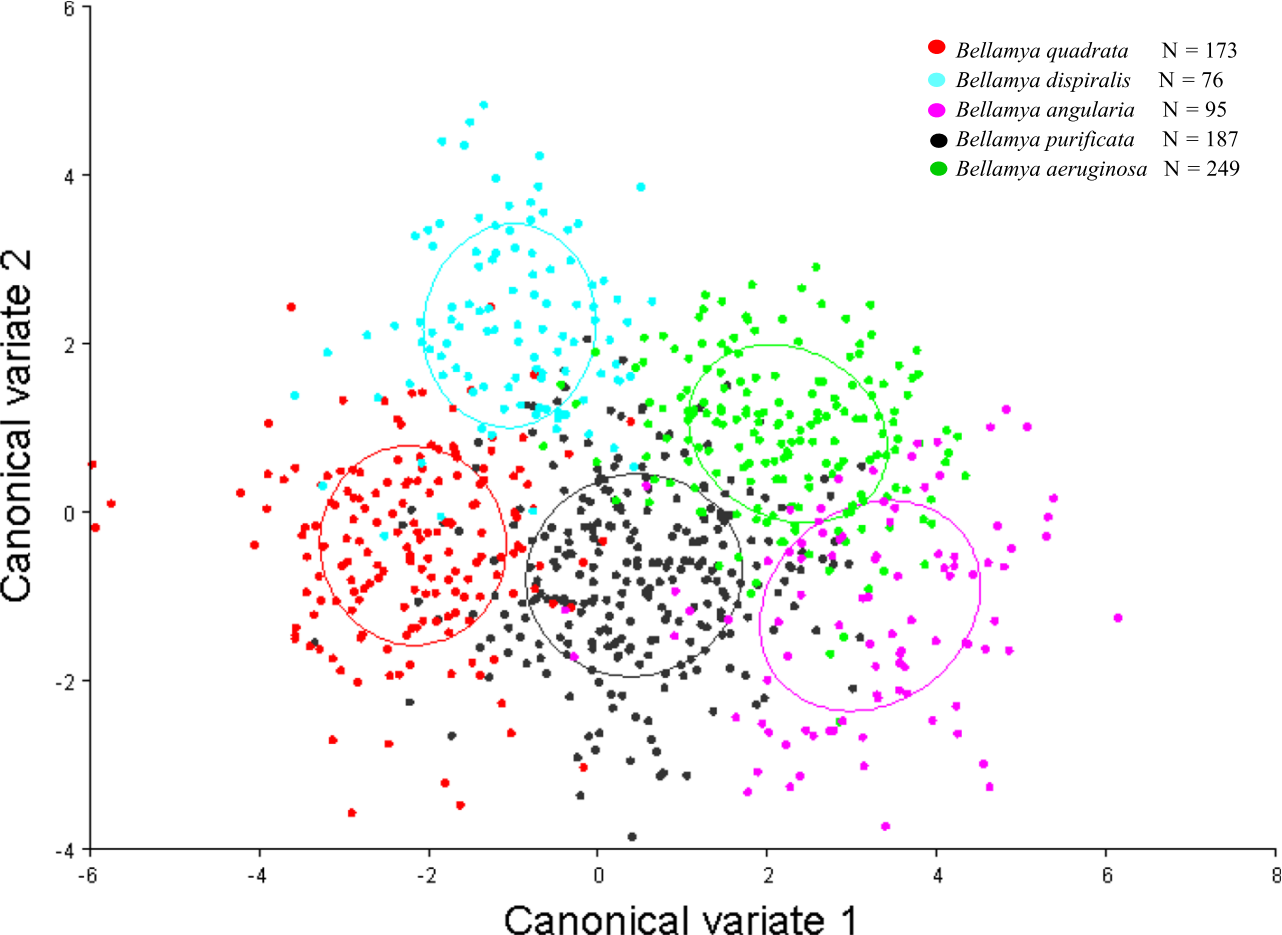
Scatter plots from the Canonical Variate Analysis (CVA) for five *Bellamya* species from China, including *Bellamya quadrata*, *B. dispiralis*, *B. angularia*, *B. purificata*, *B. aeruginosa*, and number (N) of each species was shown

Because of the low specimen number of *B. lapillorum*, *B. lapidea* and *B. turritus* (≤ 6), five *Bellamya* species (for each *N* ≥ 20), including *B. quadrata*, *B. purificata*, *B. aeruginosa*, *B. angularis* and *B. dispiralis* were used to compare the differences in shell morphology and the ultrastructure of radulae. Geometric morphometrics were used to examine shape variation through principal components analysis (PCA) and canonical variance analysis (CVA). The results showed that the first four PCs explain 60.01% of the variation in shell morphology (Additional file 1: Table S2), while the first two CVs explain 91.45% of the variation (Additional file 1: Table S3). The scatter plot of CV1 and CV2 showed clear differentiation between the five species (Fig. 1), and the results of Mahalanobis distances and Procrustes distances based on CVA showed a significant difference among the five *Bellamya* species in China (*P* < 0.01) (Additional file 1: Table S4).

The ultrastructure of radulae for the five species was examined using a scanning electron microscope (SEM, 800× magnification; Additional file 2: Figure S2a). The radulae were of stenoglossan type with a general formula of 2·1·1·1·2; each row of radula teeth consisted of one central tooth, one lateral tooth and two marginal teeth on each side (Additional file 2: Figure S2a). Comparison of the radular morphology revealed that the five *Bellaymya* species differed in their radula tooth formula, i.e. the number of central teeth, lateral teeth, inner marginal and outer marginal teeth (Additional file 1: Table S5, Additional file 2: Figure S2b).

### Sequence polymorphism

A total of 319 individuals from 16 species were sampled. Due to sequencing failure for some genes the complete dataset of all 4 genes included 133 individuals. The datasets for the individual genes were larger: the COI dataset consisting of 295 sequences with a length of 658 bp. The alignment contained 166 (25.2%) polymorphic sites, 136 (20.7%) of which were parsimony informative. The 16S dataset contained 190 sequences each 508 bp in length with 135 (26.6%) variable sites, 108 (21.3%) of which were parsimony informative. The nuclear genes were far less variable: the H3 dataset contained 159 sequences each 376 bp with 34 (9.0%) variable sites, 28 (7.4%) of which were parsimony informative. We analyzed 189 sequences for the 28S gene fragment, which were 391 bp in length and contained only 24 variable sites (6.1%), 13 (3.3%) of which were parsimony informative.

### Species tree and haplotype network

**Fig. 2:**
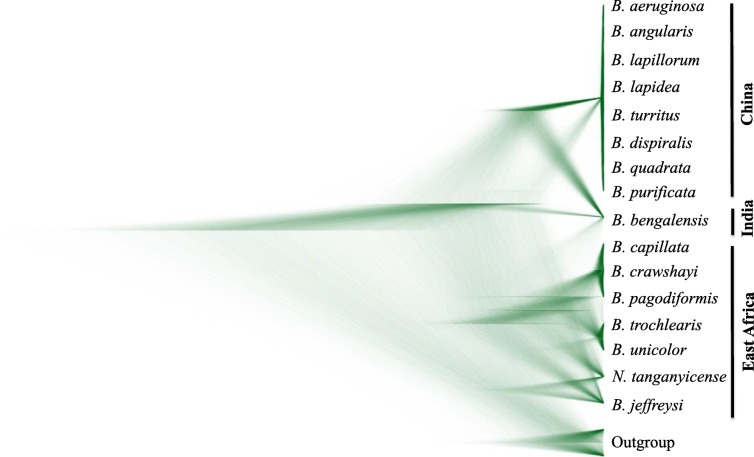
Species tree of *Bellamya* constructed by BEAST visualized with DENSITREE. DENSITREE draws all trees gained from a Bayesian phylogenetic run using transparent lines. In areas where many trees agree on a specific topology and branch length, a densely colored area will be observed

The resulting species tree showed a clear pattern of divergence between Africa and Asia (Fig. 2); all *Bellamya* species from China and East Africa formed own monophyletic clades with high support values as well as high posterior probabilities value (Additional file 3: Figure S3, ML phylogenetic). *Bellamya* species within East Africa showed a pattern of deep divergence and almost every lake formed its own unique genetic cluster. However, *Bellamya* species from China did not show any clear differentiation; different *Bellamya* species sampled from Northern to Southern parts of China formed a single genetic cluster.

The Median Joining Network showed two distinct matrilines (Additional file 4: Figure S4): one contained four main lake lineages from East Africa (Lake Vitoria, Lake Malawi, Lake Tanganyika and Lake Mweru); the second group is represented by a single highly admixed lineage from China and no differentiation could be detected.

### Phylogenetic tree construction and molecular clock estimates

**Fig. 3:**
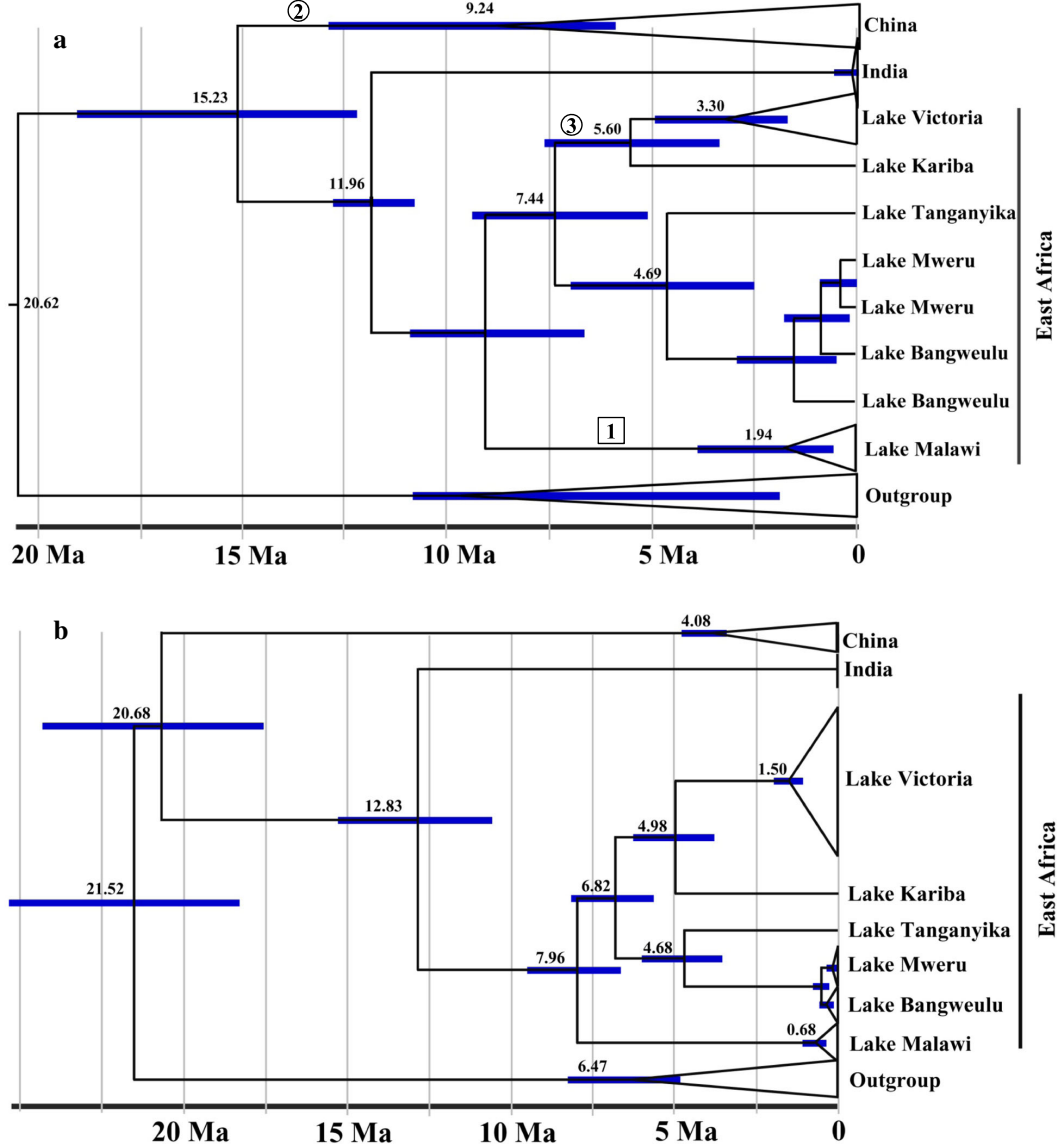
Dated phylogeny using a) geological (number in squares, 1: 1.6 Ma [112]) and fossil calibration points (number in circles, 2: 13 Ma [27]; 3: 4.2 Ma [14]) to estimate the divergence between Asia and Africa; b) using a rate calibration

Our fossil calibrated molecular clock analyses and the rate calibrated analyses yielded fairly similar results. In the fossil calibrated analysis the Chinese *Bellamya* clade diverged from the East African one about 15.23 Ma (million years ago) (95% confidence interval: 11.3–24.9 Ma) (Fig. 3a). The rate calibrated [33] analysis showed the same phylogenetic pattern and estimated the split between China and East Africa at approximately 20.68 Ma (CI: 15.3–28.2 Ma) (Fig. 3b). The divergence of the East African clades started between 8 (rate calibration) and 9 Ma (fossil calibration) matching the evolution of the Lake Tanganyika basin between 9 and 12 Ma [34]. The Chinese clade has formed between 4 (rate calibration) and 9 Ma (fossil calibration).

## Discussion

We sampled *Bellamya* species from China, India and East Africa, and employed phylogenetic and phylogeographic methods to investigate their divergence patterns. As we only included four specimens from India (*Bellamya bengalensis* IS01 ~ IS04, see Additional file 5: Table S1), their diversity pattern will not be further discussed. We found two very different monophyletic lineages, one in China, the other in East Africa. However, the patterns of divergence on both continents represented two extremes with deep splits within East Africa and no significant genetic differentiation in China. In the following, we discuss our findings in the context of the geographic and climatic histories of the continents in detail.

### Monophyletic Chinese and east African lineages

The eight *Bellamya* species collected in China can be identified according to their shell morphology characterized by Zhang and Liu (1960) [35]. Furthermore, there are significant differences in geometric characteristics of their shells and the ultrastructure of their radulae for five speceis of them.

Diversification of taxa distributed in Africa and Asia has received much attention and especially fish and snails have been extensively studied [36–38]. Our results suggest the presence of strong divergence between China and East Africa with monophyleltic clades with own evolutionary trajectories in both regions. In the East African lineage, based on molecular data, Sengupta et al. (2009) [24] reported seven putative species in accordance with taxonomic assignments [30]. In China, however, the eight species determined according to the current taxonomy could not be recovered with molecular data, and no significant differentiation between geographic populations was detected. Furthermore, we found no significant population genetic structure and phylogeographic pattern for both *B. aeruginosa* and *B. purificata* in China [39, 40].

Although two very different monophyletic lineages are found in China and East Africa, it is unknown whether the split between the Chinese and African populations is the result of vicariance or of long-distance dispersal. Many dispersal events between Africa and Asia have been reported and have been suggested to be the result of the contact between Africa and Eurasia via the Arabian Peninsula about 20–15 million years ago (Ma) [41–43]. Many species migration events between the two continents have been postulated to have occurred at this time interval (between the late Oligocene and mid Miocene) [37, 44]. The results of the molecular clock analyses suggest that the divergence of the Chinese and the African lineages of *Bellamya* also occurred at that time and hence dispersal via the Indian route seems a likely scenario.

### Phylogrographic pattern

Geological data indicates that India separated from Africa around 65 Ma [45]. It subsequently collided with Asia between 20 and 55 Ma depending on the model estimates [46–50]. Along with the uplift of Tibetan Plateau, the separation of India and Africa has created the most important biogeographical barriers that led to diversification events between Southeast Asia and India in many taxa [51], and also with other geographical regions, including the Middle East and Africa [52].

Our results suggested that East African *Bellamya* diverged from Chinese lineages during the early Miocene (approximately 20~15 Ma, Fig. 1a, b) when the Afro-Arabian plate and Eurasia became connected about 20.5–14.8 Ma [53]. The divergence time far preceded the time of the rapid uplift of Tibetan Plateau (10~8 Ma) [54] and even later (3.6~0.8 Ma) [55], and far postdated the separation between India and Africa [45], suggesting that dispersal rather than vicariance has created the current distribution pattern of *Bellamya* in Southeast Asia and East Africa. Well dated *Bellamya* fossils have been recovered along Southeast Asia [56, 57] and the Middle-East [28], as well as from Africa [58], all postdating the Gondwana fragmentation [59]. This all suggests that the overland migration hypothesis is a good candidate explaining the distribution of *Bellamya* in Africa and Asia. The hypothesis of ‘transoceanic long distance dispersal’, proposed by Gittenberger (2012) [60], however, is a good alternative. Our data suggest that a Miocene dispersal event from Asia to Africa, through a broad connection between the Arabian Peninsula and Ethiopia [42, 61], was much more likely than an ancient Gondwanan vicariance based on the relatively low genetic diversity between the clades in Africa [14], while middle to high genetic diversity in COI was found in China [39, 40].

A strikingly similar pattern has been inferred from a phylogeny constructed for the family Viviparidae: the African viviparids are the sister group to a clade comprising Asian species (including *Bellamya*), and a similar dispersal route from Asia to Africa through the connection between Yemen and Ethiopia during middle and late Miocene was concluded [14]. Particularly, during the period of 10–8 Ma (late Miocene), sea levels dropped 60 m below modern levels, connecting Africa and the Arabian Peninsula by land bridges at either end of the Red Sea and facilitating the migration to Africa [62]. *Bellamya* entered Southeast Africa around 9 Ma (Fig. 1a, b), and then dispersed to different water systems of East Africa, and continued to differentiate during Late Miocene and Pleistocene.

The Miocene seems to be the period during which *Bellamya* likely migrated from Asia for two reasons: Firstly, *Bellamya* fossils uncovered from Thailand [28] were dated at approximately 13 Ma [27], which is earlier than the split time (9.18 ~ 7.96 Ma, Fig. 1a, b) of *Bellamya* in Africa, and also much earlier than any African fossils which were dated to the Early Pliocene, ∼4.2 Ma [14] and that of Levant from the Pliocene-Pleistocene [29]; secondly, the climate during the Miocene was humid and warm, which allowed the rain forest to extent much further southward than today creating suitable habitat [63–65] for *Bellayma*, as well as for other gastropod species [24, 66, 67]. This was further corroborated by other taxa including fish and frogs [37, 41, 68].

However, the uprising of the Himalayan Mountain [54] and consequential climatic changes in the region started to impede species exchange during the Pleistocene, when the climate became much drier and cooler [69, 70]. This had caused a retreat of rainforest cover which was replaced by savannahs and deserts in Southeast Asia [57]. These unfavorable climatic conditions made a subsequent dispersal of many freshwater organisms almost impossible. Consequently, this dry period may have initiated the isolation between African and Asian populations and led to the establishment of the genetically unique species in China and East Africa (Additional file 3: Figure S3 and Additional file 4: Figure S4).

The molecular phylogeny and fossil data strongly suggested that *Bellamya* originated in Southeast Asia. However, it was still in question how this genus invaded East Africa. It has been suggested that some freshwater gastropods, such as Pachychilidae, colonized Madagascar by trans-oceanic dispersal [71]. However, this type of dispersal was generally considered as unlikely due to large intercontinental distances and salinity intolerance of many invertebrates [72]. Yet, transportation of eggs or larvae by waterfowl remains a viable dispersal route. However, colonization on terrestrial routes appears more plausible. A possible migration route would have been through India and the Arabian Peninsula (Additional file 6: Figure S5), when Africa and Arabia were in close contact during the Miocene [73]. Certainly, this migration route should be further investigated by including additional specimens and sampling locations across the Asia, India, the Arabian Peninsula and Africa in the future, as well as more *Bellamya* fossils. Similar species exchanges have been demonstrated for other organisms at that time [73–76]. The increasingly arid conditions on the Arabian Peninsula between 7 and 8 Ma [74] may have restricted dispersal subsequently, further accelerating the independent diversification of the East African and Chinese *Bellamya*.

### Contrasting patterns of divergence in Chinese and east African lineages

It is well established that distribution patterns of aquatic biota often correlate with the geomorphological features of a region and its geologic history [77–79]. We observed contrasting divergence patterns in the monophyletic *Bellamya* lineages of China and East Africa. These patterns were likely associated with the divergent climatic and geological histories of the two regions. The collision between the Indian and Asian plates [46] had uplifted the Qinghai-Tibetan Plateau from around 1000 m to 4000–5000 m above sea level approximately in the middle and late Miocene and even more recently [54, 55]. This collision had caused profound changes of the trajectories of many major rivers and geomorphological features in this region [55, 80]. The upper Yangtze River originally drained into the South China Sea through the Paleo-Red River until it was captured by the middle and lower reaches of the Yangtze River and reversed its route to flow into the East China Sea [81, 82]. The resulting river capture and flow reversal events, in turn, likely had a dramatic effect on the evolution of many aquatic organisms, including *Bellamya*. These events might have promoted genetic exchange between previously isolated *Bellamya* lineages, and would have resulted in range expansions of lineages which may previously have evolved in isolation removing any geographic signatures. Many tributaries flowed into the main river channel and numerous lakes interlace with them and formed a complete riverine–lacustrine network [83]. The newly established connectivity likely facilitated gene flow between different *Bellamya* populations, supported by the medium to high migration rates found in the *B. aeruginosa* across China [39]. Furthermore, the flooding, anthropogenic translocations and animal-mediated dispersal via waterfowl [84] would also expedite gene flow and obscure phylogeographic patterns in China [39, 40]. Our analyses indicated that Chinese lineages of *Bellamya* had an onset of divergence estimated within the last 9 to 4 Ma. This estimate might even be inflated by the presence of ancestral polymorphisms and the actual age of many lineages might be even younger [85]. This is supported by the young age of most *Bellamya* fossil recovered from China, most of which were from the Holocene and Early Pleistocene [86–88]. Therefore, the *Bellamya* lineages in China, have not had sufficient time to accumulate mutations. Furthermore, gene flow between geographic populations and the possible occurrence of hybridization between sympatric species, which could not complete their lineage sorting, may have led to the observed lack of phylogenetic resolution in our data.

Similarly, East Africa underwent dramatic climatic and geological changes at the same period. Over the past few million years, the constant expansion and regression of the great East African lakes have led to the repeated formation and loss of habitats [89]. This landscape dynamics had dramatic effects on the organisms inhabiting in the lakes, including *Bellamya* [58]. However, these events caused an opposite effect on *Bellamya* evolution compared to their Chinese counterparts. Most of the East African lakes harbor their own endemic *Bellamya* lineages [24], which were also confirmed in our phylogenetic analysis. These lineages are most likely the results of relatively strong isolation among these water bodies. Intralacustrine speciation could also be explained by the frequent water level fluctuations causing high speciation rates and driving rapid diversification [90]. The African mega-droughts caused strong water level changes and probably led to strong bottlenecks or even extinctions for many organisms living in the shallow parts of the lakes. Lake Malawi, for example, was much shallower than today before ~ 0.8 Ma and experienced larger environmental fluctuations during that time [3]. The lake regression created empty ecological niches allowing for a secondary species radiations when the climate became favorable again [25]. The divergence time of the Lake Malawi lineage was dated at around 0.68 Ma, consistent with the refilling of the basin [72] and the radiation of another gastropod *Lanistes* in the lake [91].

## Conclusions

*Bellamya* represents an ideal system to test how past climatic and geological events impacted current biodiversity. Molecular phylogenetics indicate that *Bellamya* in China and East Africa represent genetically unique phylogenetic clades. Our molecular dating suggested that the *Bellamya* in East Africa diverged from China approximately 20 Ma, suggesting that dispersal rather than vicariance was responsible for the extant biogeographic pattern of *Bellamya* in Africa and Asia. The different divergence patterns of *Bellamya* on the different continents are mainly attributed to the contrasting climatic and geological events.

## Methods

### Sample collection

**Fig. 4:**
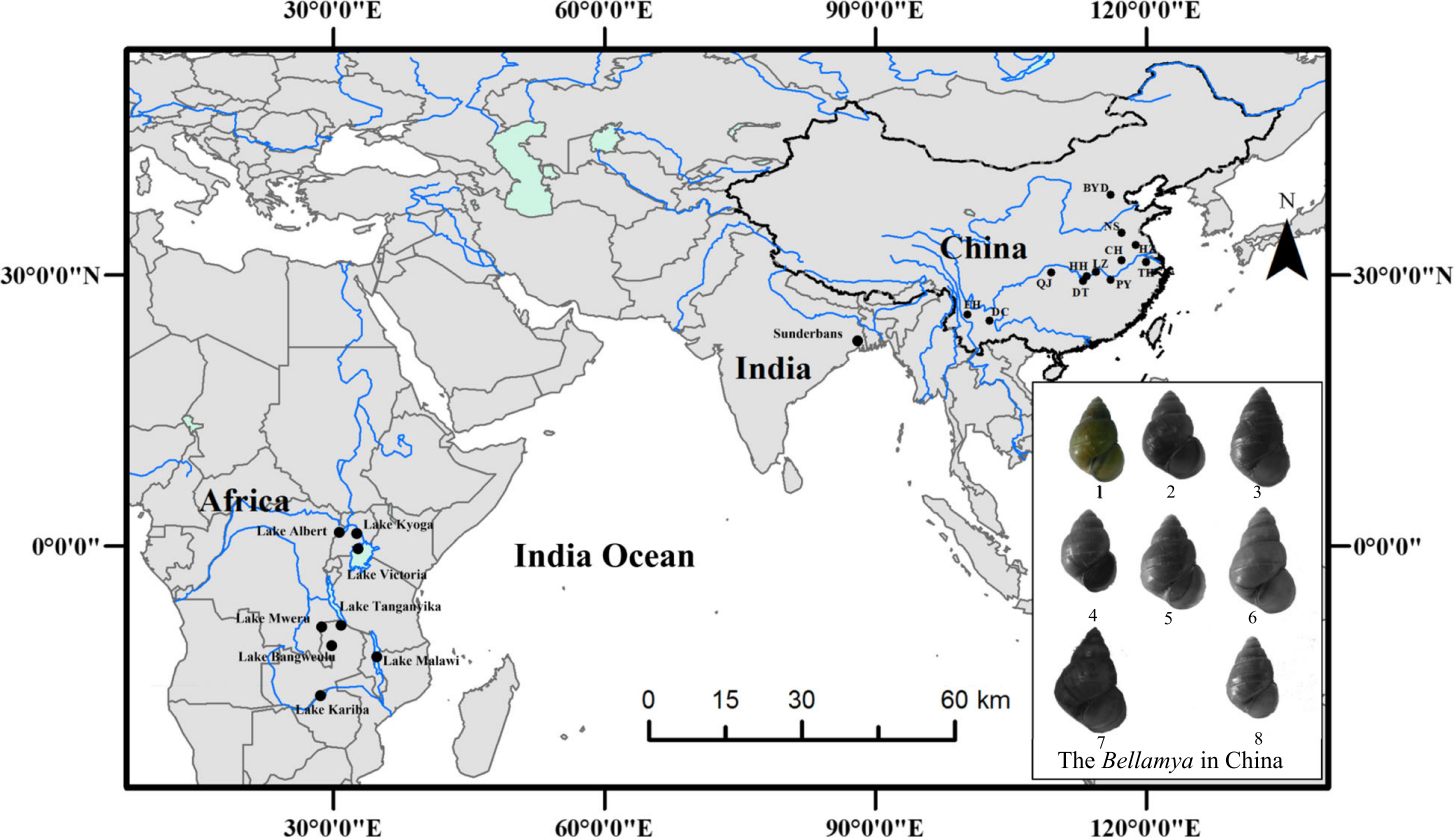
Sampling localities of *Bellamya* in China, India and Africa, the blue lines represent the river system; the black solid circles mean the sampling location: BYD (Baiyangdian), NS (Nanshihu), HZ (Hongzehu), TH (Yixing Taihu), CH (Chaohu), PY (Poyanghu), LZ (Liangzihu), HH (Honghu), DT (Dongtinghu), QJ (Qingjiang), EH (Erhai), DC (Dianchi); 1 - *B. aeruginosa*, 2 - *B. purificata*, 3 - *B. quadrata*, 4 - *B. angularis*, 5 - *B. lapillorum*, 6 - *B. dispiralis*, 7 - *B. turritus*, 8 - *B. lapidea*

*Bellamya* is a genus of freshwater snails, belonging to family Viviparidae. It is widely distributed across Asia, India and Africa. Currently, 18 endemic species are described both in China [32] and Africa [24]. Here, we followed the species identifications which were described by Zhang and Liu (1960) [35], and collected eight common species of *Bellamya* from several lakes across China (Additional file 5: Table S1, Fig. 4).

In addition, published *Bellamya* sequences from species outside of China were obtained from GenBank. Overall, we obtained a balanced sampling with 8 described taxa sampled in China and seven from East Africa, while just one species from India. A complete overview of the taxon sampling is provided in Additional file 5: Table S1.

In order to test for morphometric difference of *Bellamya* in China, ensuring the reliability of classification, shell geometric morphometrics was used to examine shape variation through principal components analysis (PCA) and canonical variance analysis (CVA) in MorphoJ v. 2.0 [92]. Shells of *Bellamya* were photographed with a Nikon CoolPix 4500 and 19 landmarks (Additional file 7: Figure S1) were determined; X and Y coordinates were recorded according to Minton and Wang (2011) [93] by the software TpsDig2 [94]. For each specimen, the X and Y landmark coordinates were translated to the origin, rotated and scaled by the Generalized Procrustes Analysis [95] in TpsRelwarp [96] to remove non-shape variations. The specimen number of each species was more than 70 (the number of each species was shown in Fig. 1) in this study.

Furthermore, the radulae were investigated and compared using light and scanning electron microscope. The radulae were extracted from the snail buccal cavity using a stereomicroscope, and ten radulae of each species were examined. The radulae were first boiled in 5% sodium hydroxide for 5 min, and then washed in an ultrasonic scrubber for 2 min. Dehydration was performed by immersing the radulae in increasing alcohol concentrations (10, 30, 50, 70, 80 and 95%, respectively). Subsequently, the specimens were mounted on stubs with the help of a sharp-tipped needle on carbon conductive adhesive tapes. The stubs were then coated with gold and observed using a scanning electron microscope (AMRAY-1000B).

### DNA extraction, sequencing and molecular analyses

Total genomic DNA was isolated from foot muscle tissue using the DNeasy kit following the manufacturer’s protocol (QIAGEN DNeasy® Blood and Tissue Kit, Shanghai, China). DNA was subsequently quantified with a spectrophotometer (NanoDrop 2000, Thermo Fisher Scientific, America). Four gene fragments were amplified by polymerase chain reaction (PCR): two mitochondrial genes (Cytochrome Oxidase I (COI) and 16S rRNA) and two partial nuclear genes (Histone H3 and 28S rRNA). COI, 16S and H3 were amplified using primers provided in the literature (COI - LCO1490 (forward primer) and HC02198 (reverse primer) [97], 16S - 16sar-L and 16sbr-H [98], and H3 - F (forward) and R (reverse) [99]). For 28S a new primer pair was designed with Primer Premier 5.0 (Premier Biosoft International, Palo Alto, California, USA) using various mollusk sequences deposited in GenBank (FJ405581-FJ405634): forward primer 5′-CCGCTGAATTTAAGCATATCACT-3′ and reverse primer 5′-CGGTTTCACGTACTCTTGAACTC-3′. PCR was conducted in 50 μl volume reactions using the PrimeSTAR® HS DNA Polymerase (TAKARA, Dalian in China), 5 × PrimerSTAR® Buffer (Mg^2+^ plus), 10 μl; dNTP Mixture (each 2.5 mM), 4 μl; Primers, each 1 μl; DNA, 1 μl; PrimeSTAR® HS DNA Polymerase (2.5 U/μl), 0.5 μl, purified water up to 32.5 μl. Thermal cycling conditions were as follows: 94 °C for 4 min, 35 cycles of 98 °C for 10 s, annealing for 5 s and 72 °C for 45 s; cycling was terminated by 8 min of final extension at 72 °C. The annealing temperatures were 50 °C, 48 °C, 54 °C, and 52 °C for COI, 16S, H3 and 28S, respectively. PCR products were purified using the High Pure Product Purification Kit (TAKARA). Cycle-sequencing reactions were performed using BigDye terminator v. 3.1 and analyzed using an ABI-PRISM 3730 sequencer at Sangon Biotech (Shanghai, China). Both DNA strands were sequenced for each of the gene fragments. Sequences were edited and aligned using both strands for confirmation with Geneious v. 5.6.5 [100]. All sequences were aligned with MAFFT [101] as implemented in Geneious and compared to available sequences in GenBank to confirm validity. The sequences were submitted to GenBank (accession numbers (KF535211-KF535893); details provided in Additional file 5: Table S1).

### General statistics and phylogenetic analyses

General alignment statistics were calculated with DNASP v.5 [102]. To test our first hypothesis that samples from both continents represent monophyletic lineages and to estimate a species tree, we constructed a phylogeny analyzing all four genetic markers simultaneously using the coalescent-based approach implemented in *beast [103]. In the species tree analysis we included all 319 individuals (Additional file 5: Table S1). *Viviparus ater*, *V. contectus* and *Neothauma tanganyicense* were used as outgroups. The best-fitting substitution models for each gene fragment were determined by JMODELTEST v.2.1.4 [104]. The GTR + I + G model was chosen for COI and H3. The GTR + I model was determined to be most suitable for 28S. The GTR + G model was selected for the 16S. An input file for *beast was generated using the program BEAuti v.1.7.4 [105]. The Yule process was chosen as the tree prior as suggested to be useful for species level data [106] and the population size prior was set to constant. The analysis was run for 100 million generations to ensure convergence; trees were sampled every 10,000 generations. Convergence was checked in tracer v1.7.1 [107] and inferred through the effective sample sizes (ESS) of parameters, which were all above 200. The first 1000 saved trees (10%) were discarded as burn-in after ensuring the likelihood scores reached a plateau which was determined with the program tracer v1.7.1 [107]. Trees were visualized in figtree 1.4 [108] displaying posterior probabilities as branch support. Posterior probabilities above 95% were considered as evidence for substantial support at a node. A consensus tree was generated using TREEANNOTATOR v.1.6.1 [105] discarding a burn-in of 10%. We further used densitree 2.01 [109] to visualize the species tree. Densitree draws all trees gained from a *beast run using transparent lines. In areas where many trees agree on a specific topology and branch length, a densely colored area will be observed.

We also constructed a phylogenetic tree with the COI dataset using Maximum Likelihood (ML) and Bayesian analysis; here only COI was used, as almost all individuals were successfully sequenced for this gene. We tested the COI data for saturation using DAMBE v.5 [110]. The test revealed an Iss value that was significantly lower than the Iss.c in all cases (*P* < 0.0000), indicating the suitability of the COI data for phylogenetic analysis. The GTR+ G substitution model fitted the data best and was employed for the COI dataset. The ML tree was computed using RAxML 8.0 [111] with 1000 bootstrap replications to obtain branch support. A partitioned Bayesian analysis was conducted using MrBayes 3.2.2 [112] (Ronquist et al., 2012). Mixing of the MCMC chains of the two independent runs was monitored with TRACER v1.7.1 [107] and the analysis was terminated after the average standard deviation of the split frequencies fell under 0.01. The first 10% of the sampled approximately 10 million generations were discarded as burn-in. The final trees were visualized in figtree 1.4 [108].

To test our second hypotheses that several geographic lineages endemic to specific regions of Southeast Asia exist, we generated a haplotype network for the COI data (including all samples) and colored each haplotype by the geographic region from which it was collected. An unrooted network was constructed using network 4.6.0.0 and NETWORK PUBLISHER 1.3.0.0 (http://www.fluxus-engineering.com/sharenet.htm) applying the median-joining and Maximum Parsimony options.

To test the third hypothesis that *Bellamya* originated in Africa and dispersed to Asia rather than a split by vicariance, divergence times were estimated using a molecular clock approach as implemented in *BEAST. We used the two mitochondrial genes (COI and 16S) and two nuclear genes (H3 and 28S) in this analysis (133 sequences) and employed a ‘relaxed clock model’ with two calibration points. The first calibration point was the timing of the East African mega-drought between 1.6–1.8 Ma, which almost desiccated Lake Malawi completely. The refilling of the lake about 1.6 Ma was utilized as one calibration point for the Lake Malawi lineage [72]. The node was calibrated using a normal distribution with a mean at 1.6 Ma and a standard deviation of 1 Ma. *Bellamya* fossil data was used as the second calibration point; fossil records were obtained from (1) Vichaidid et al. (2007) [27] from the Mae Moh basin, Thailand, which dates at approximately 13 Ma; (2) Van Bocxlaer et al. (2008) [14], and the Turkana basin in Kenya, dated at approximately 4.2 Ma. The analysis was run for 100 million generations logging trees every 10,000 generations. Convergence was checked with Tracer. Node ages are displayed together with 95% highest posterior density bars indicating a range of age estimates. We performed a second molecular clock analyses with similar settings, but using a rate calibration. For this we used only the COI gene with a strict clock and a publishedrate of 0.0130 per Ma [33]. *Viviparus ater* (FJ405882), *V. contectus* (FJ405835) and *Neothauma tanganyicense* (FJ405843) served as outgroups for both analyses.

## Additional files

Additional file 1: **Table S2.** Eigenvalues, percentage of variance and cumulative percentage of the first four principle components. **Table S3.** Eigenvalues, percentage of variance and cumulative percentage of the four canonical variate axes. **Table S4.** Differences based on geometric morphometrics of shell shape among five *Bellamya* species in China. Mahalanobis and Procrustes distances computed from the Canonical Variate analysis, *P*-values for the significance of the interspecies distances were computed using permutation tests (10,000 replications); all *P* < 0.0001. **Table S5.** Difference of radula ultrastructure of five *Bellamya* species in China, details in Additional file 7: Figure S1. (DOCX 21 kb)

Additional file 2: **Figure S2.** a) Scanning electron micrographs of the radulae of *Bellamya*; C, central teeth; L, lateral teeth; IM, inner marginal teeth; OM, outer marginal teeth; Scale bar = 100 μm. b) Radulae morphology of 5 *Bellamya* species. A, central tooth; B, lateral tooth; C, inner marginal tooth; D, outer marginal tooth; 1, *B. aeruginosa*; 2, *B. purificata*; 3, *B. quadrata*; 4, *B. angularis*; 5, *B. dispiralis*; Scale bar = 20 μm. (PPTX 663 kb)

Additional file 3: **Figure S3.** Phylogenetic tree of *Bellamya* generated from COI sequences constructed by RAxML 8.0. Numbers above the branches represent bootlstrap values (> 50%) for maximum likelihood estimations and the posterior probability (Bayesian inference, BI > 0.50) calculated by Mrbayes. (PDF 606 kb)

Additional file 4: **Figure S4.** Median-joining haplotype network of 292 COI sequences. The size of the circles represents haplotype frequency. Each connecting line represents a single nucleotide substitution. Blue circles represent haplotype group 1 (Southeast Asia, numbers H38–138); red circles show haplotype group 2 (East Africa, numbers H1–37, except H16 and H37). In group 2, different colours represent different lake populations, Lake Malawi (red); Lake Victoria (rose red); Lake Kariba (purple); Lake Mweru (orange); Lake Tanganyika (green); Lake Bangwerulu (black); yellow represents Indian specimens (H16 and H37). For sampling details please see Fig. 4 and Additional file 5: Table S1. (PPTX 498 kb)

Additional file 5: **Table S1.** Taxon sampling sites, sequence IDs, and GenBank accession numbers used in this study. (DOCX 81 kb)

Additional file 6: **Figure S5.** Inference of historical dispersion route and schematic view of the extant fossil record locations for *Bellamya*. Blue, orange and red indicate the possible distribution range of *Bellamya* in Asia, India and Africa. Arrows indicate the possible dispersion routes. Data were obtained from (1) Huang et al. (2007), Yixing, Jiangsu Province, China; (2) Wang (1983), Zhenpiyan, Guilin Province, China; (3) Vichaidid et al. (2007), Mae Moh basin, Thailand; (4) Ashkenazi et al. (2010), Gesher Benot Ya’aqov, Israel; (5) Sivan et al. (2006), Erq el-Ahmar, Israel; (6–10) Van Bocxlaer et al. (2008) and references therein. (PPTX 364 kb)

Additional file 7: **Figure S1.** Positions of 19 landmarks superimposed on a photograph of *Bellamya*. (PPTX 400 kb)

## Abbreviations

16S: 16S ribosomal RNA
28S: 28S ribosomal RNA
COI: Cytochrome C Oxidase Subunit I
ESS: The effective sample sizes
H3: Histone H3
Ma: Million years
MJ network: Median Joining Network
