# Supplementary material for: Phylogeography of Bellamya (Mollusca: Gastropoda: Viviparidae) snails on different continents: contrasting patterns of diversification in China and East Africa
Source: BMC Evol Biol. 2019 Mar 21;19:82. doi: 10.1186/s12862-019-1397-0 (PMC6429760; doi:10.1186/s12862-019-1397-0)
Supplement: Supplementary file 1 — Table S2. Eigenvalues, percentage of variance and cumulative percentage of the first four principle components. Table S3. Eigenvalues, percentage of variance and cumulative percentage of the four canonical variate axes. Table S4. Differences based on geometric morphometrics of shell shape among five Bellamya species in China. Mahalanobis and Procrustes distances computed from the Canonical Variate analysis, P-values for the significance of the interspecies distances were computed using permutation tests (10,000 replications); all P < 0.0001. Table S5. Difference of radula ultrastructure of five Bellamya species in China, details in Additional file 7: Figure S1. (DOCX 21 kb) [file 12862_2019_1397_MOESM1_ESM.docx]

Table S2 Eigenvalues, percentage of variance and cumulative percentage of the first four principle components.

| Principle  components | Eigenvalues | % variance | Cumulative% Variance |
| --- | --- | --- | --- |
| PC1 | 0.00080504 | 32.573 | 32.573 |
| PC2 | 0.00031209 | 12.628 | 45.201 |
| PC3 | 0.00019456 | 7.872 | 53.073 |
| PC4 | 0.00017147 | 6.938 | 60.011 |
| … | … | … | … |

Table S3 Eigenvalues, percentage of variance and cumulative percentage of the four canonical variate axes.

|  | Eigenvalues | % Variance | Cumulative % |
| --- | --- | --- | --- |
| CV1 | 3.55653359 | 70.464 | 70.464 |
| CV2 | 1.06373894 | 21.075 | 91.539 |
| CV3 | 0.25488008 | 5.05 | 96.589 |
| CV4 | 0.1721743 | 3.411 | 100 |

Table S4 Differences based on geometric morphometrics of shell shape among five *Bellamya* species in China. Mahalanobis and Procrustes distances computed from the Canonical Variate analysis，P-values for the significance of the interspecies distances were computed using permutation tests (10000 replications); all *P* < 0.0001.

|  | *B. quadrata* | *B. angularis* | *B. purificata* | *B. dispiralis* | *B. aeruginosa* |
| --- | --- | --- | --- | --- | --- |
| Mahalanobis distances among species: | | | |  |  |
| *B. quadrata* |  |  |  |  |  |
| *B. angularis* | 5.5628 |  |  |  |  |
| *B. purificata* | 4.6501 | 2.7895 |  |  |  |
| *B. dispiralis* | 2.9918 | 5.5088 | 3.7471 |  |  |
| *B. aeruginosa* | 2.8309 | 3.3371 | 2.6676 | 3.4133 |  |
| Procrustes distances among species: | | | |  |  |
| *B. quadrata* |  |  |  |  |  |
| *B. angularis* | 0.0756 |  |  |  |  |
| *B. purificata* | 0.0608 | 0.0332 |  |  |  |
| *B. dispiralis* | 0.0281 | 0.0843 | 0.0602 |  |  |
| *B. aeruginosa* | 0.0526 | 0.0294 | 0.0182 | 0.0584 |  |

Table S5 Difference of radula ultrastructure of five *Bellamya* species in China, details in Fig. S1.

| Species | dentition formula | | | |
| --- | --- | --- | --- | --- |
|  | central teeth | lateral teeth | inner marginal teeth | outer marginal teeth |
|  | No. denticles/ formula | No. denticles/ formula | No. denticles/ formula | No. denticles |
| *B. quadrata* | 9 4-1-4 | 7 3-1-3 | 7 3-1-3 | 13-14 |
| *B. purificata* | 9 4-1-4 | 9 4-1-4 | 9 4-1-4 | 14-15 |
| *B. aeruginosa* | 11 5-1-5 | 11 5-1-5 | 9 4-1-4 | 10-12 |
| *B. angularis* | 9 4-1-4 | 9 4-1-4 | 7 3-1-3 | 11-13 |
| *B. dispiralis* | 7 3-1-3 | 9 4-1-4 | 7 3-1-3 | 11-13 |
