# Supplementary figures and images for: Phylogeography of Bellamya (Mollusca: Gastropoda: Viviparidae) snails on different continents: contrasting patterns of diversification in China and East Africa
Source: BMC Evol Biol. 2019 Mar 21;19:82. doi: 10.1186/s12862-019-1397-0 (PMC6429760; doi:10.1186/s12862-019-1397-0)

## Slide 1
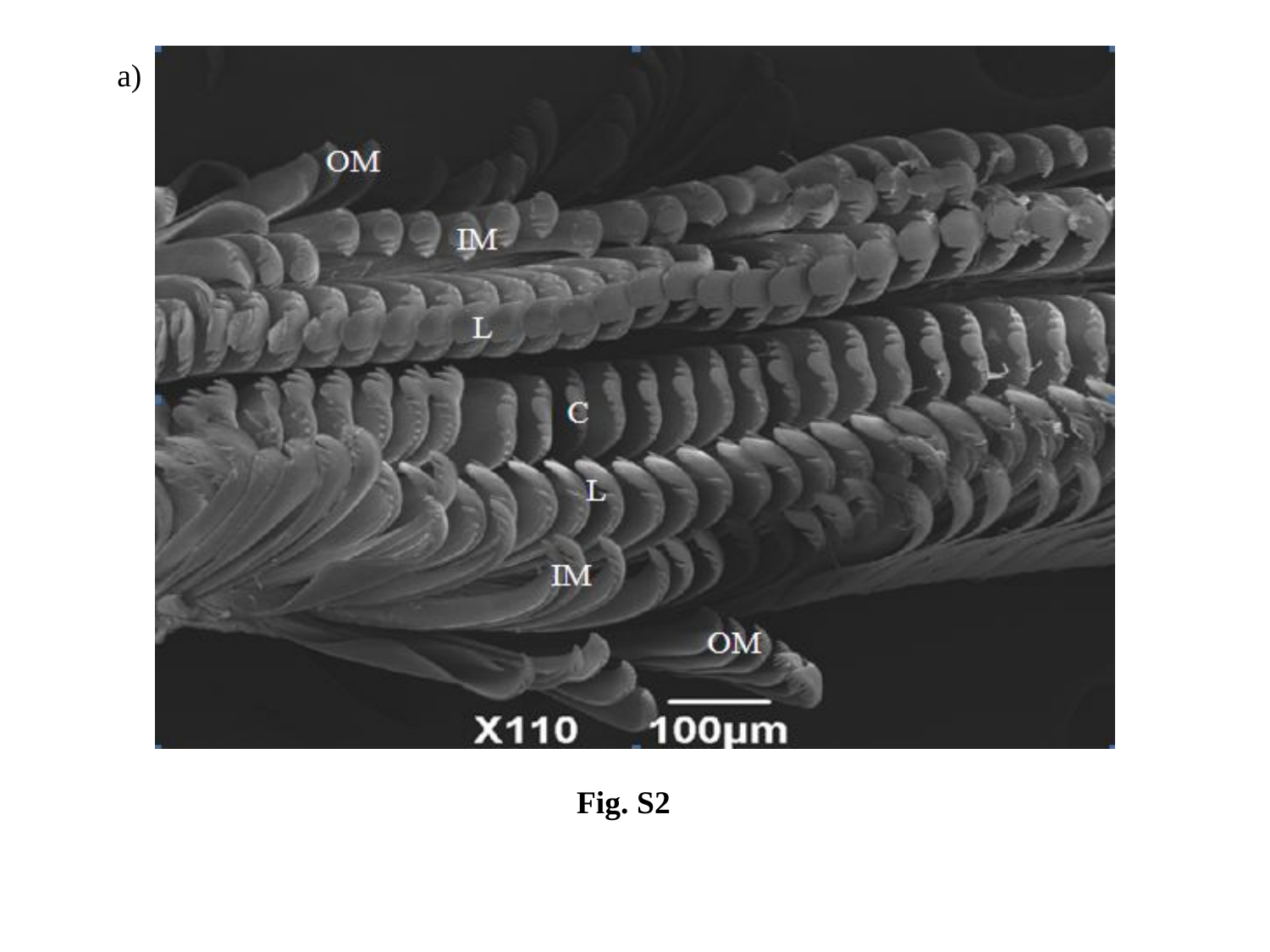

a)
Fig. S2

## Slide 2
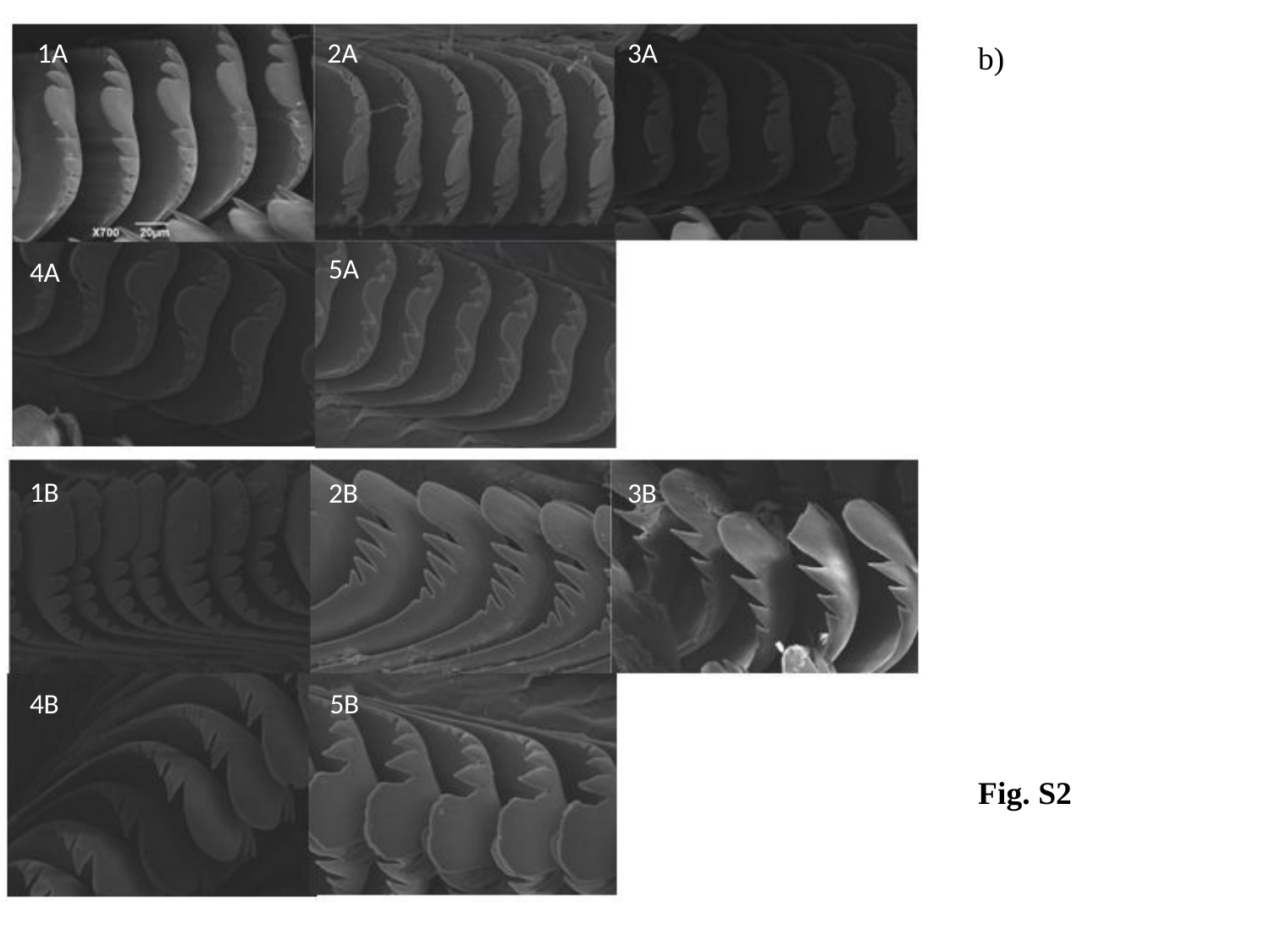

1A
2A
3A
b)
5A
4A
1B
2B
3B
4B
5B
Fig. S2

## Slide 3
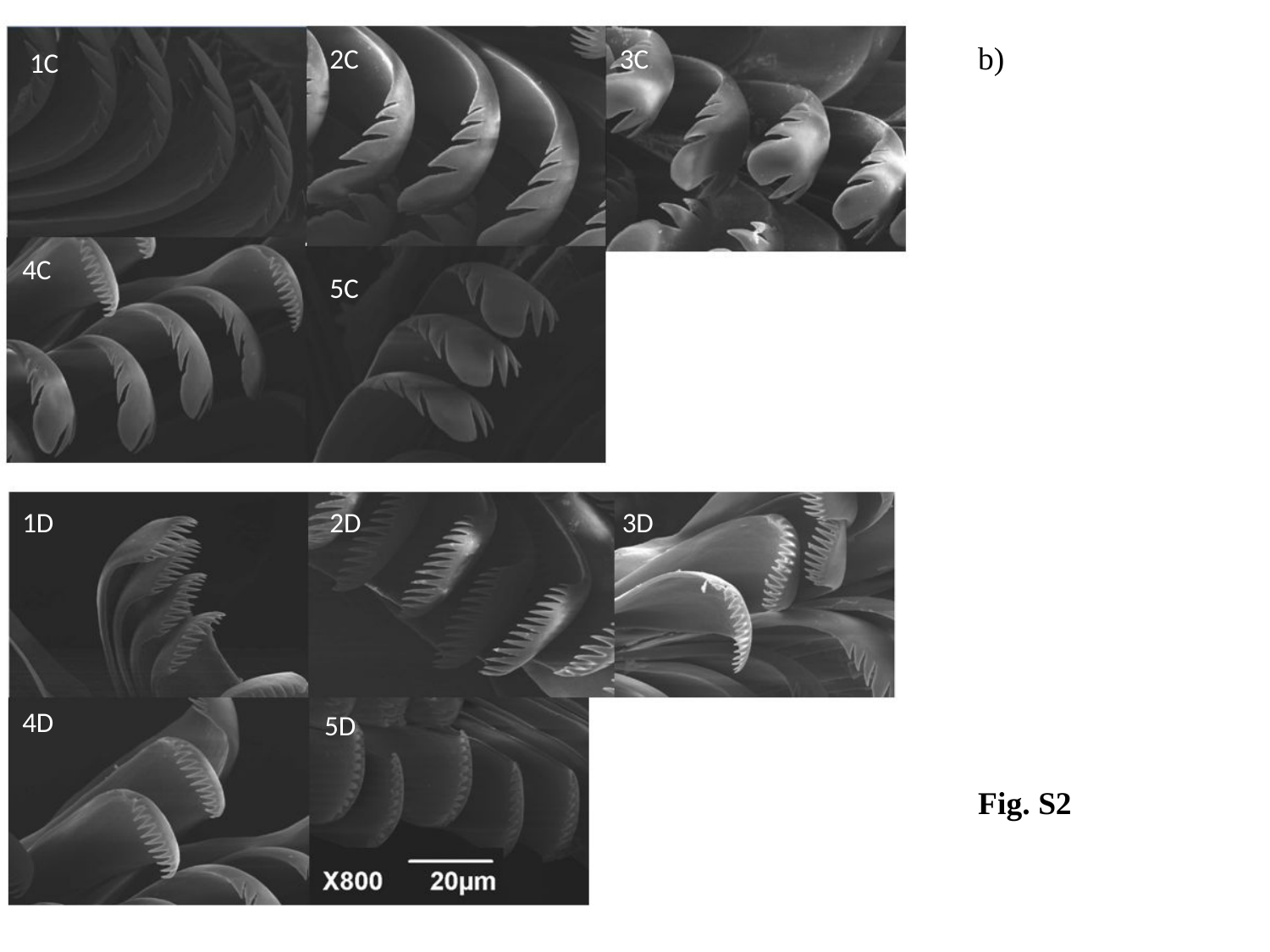

b)
2C
3C
1C
4C
5C
1D
2D
3D
4D
5D
Fig. S2

Supplement: Supplementary file 2 — Figure S2. a) Scanning electron micrographs of the radulae of Bellamya; C, central teeth; L, lateral teeth; IM, inner marginal teeth; OM, outer marginal teeth; Scale bar = 100 μm. b) Radulae morphology of 5 Bellamya species. A, central tooth; B, lateral tooth; C, inner marginal tooth; D, outer marginal tooth; 1, B. aeruginosa; 2, B. purificata; 3, B. quadrata; 4, B. angularis; 5, B. dispiralis; Scale bar = 20 μm. (PPTX 663 kb) [file 12862_2019_1397_MOESM2_ESM.pptx]

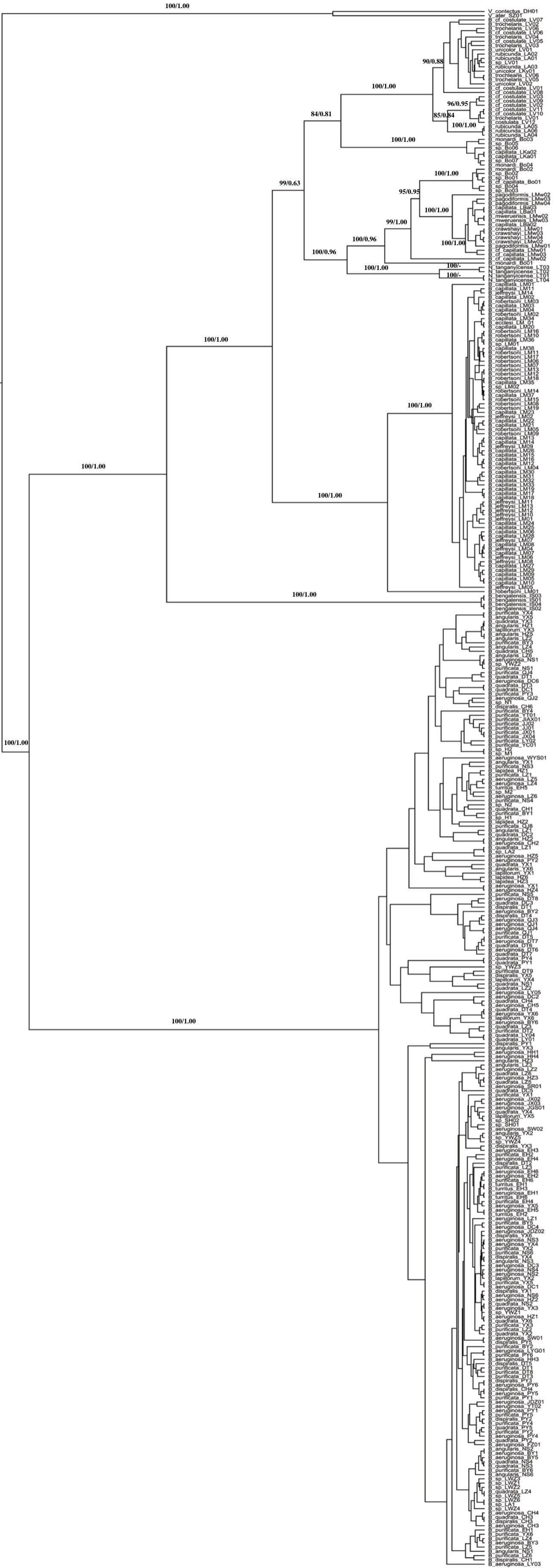

Supplement: Supplementary file 3 — Figure S3. Phylogenetic tree of Bellamya generated from COI sequences constructed by RAxML 8.0. Numbers above the branches represent bootlstrap values (> 50%) for maximum likelihood estimations and the posterior probability (Bayesian inference, BI > 0.50) calculated by Mrbayes. (PDF 606 kb) [file 12862_2019_1397_MOESM3_ESM.pdf]

## Slide 1
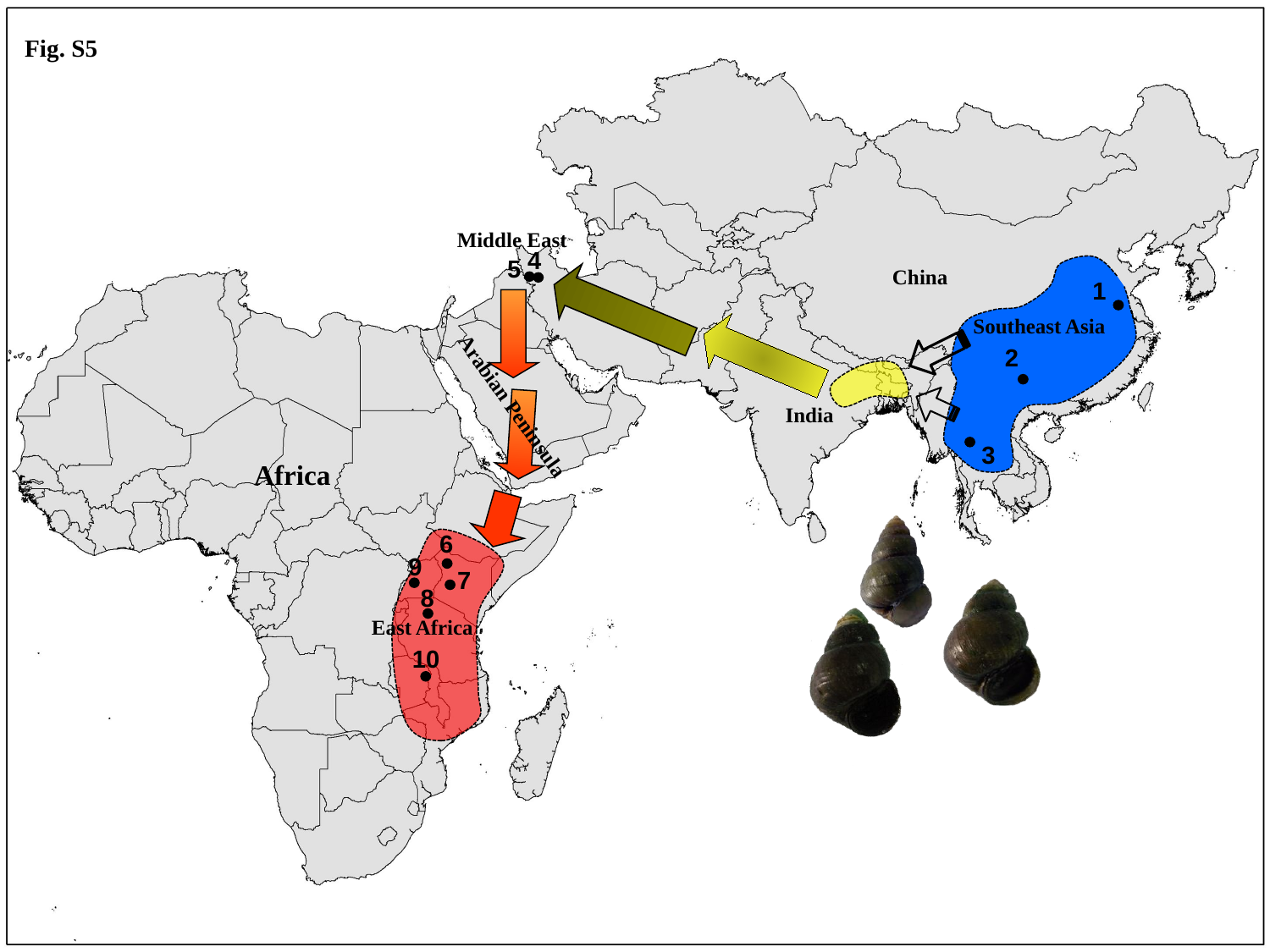

Fig. S5
Middle East
4
5
China
1
Southeast Asia
2
India
Arabian Peninsula
3
Africa
6
9
7
8
East Africa
10

Supplement: Supplementary file 6 — Figure S5. Inference of historical dispersion route and schematic view of the extant fossil record locations for Bellamya. Blue, orange and red indicate the possible distribution range of Bellamya in Asia, India and Africa. Arrows indicate the possible dispersion routes. Data were obtained from (1) Huang et al. (2007), Yixing, Jiangsu Province, China; (2) Wang (1983), Zhenpiyan, Guilin Province, China; (3) Vichaidid et al. (2007), Mae Moh basin, Thailand; (4) Ashkenazi et al. (2010), Gesher Benot Ya’aqov, Israel; (5) Sivan et al. (2006), Erq el-Ahmar, Israel; (6–10) Van Bocxlaer et al. (2008) and references therein. (PPTX 364 kb) [file 12862_2019_1397_MOESM6_ESM.pptx]

## Slide 1
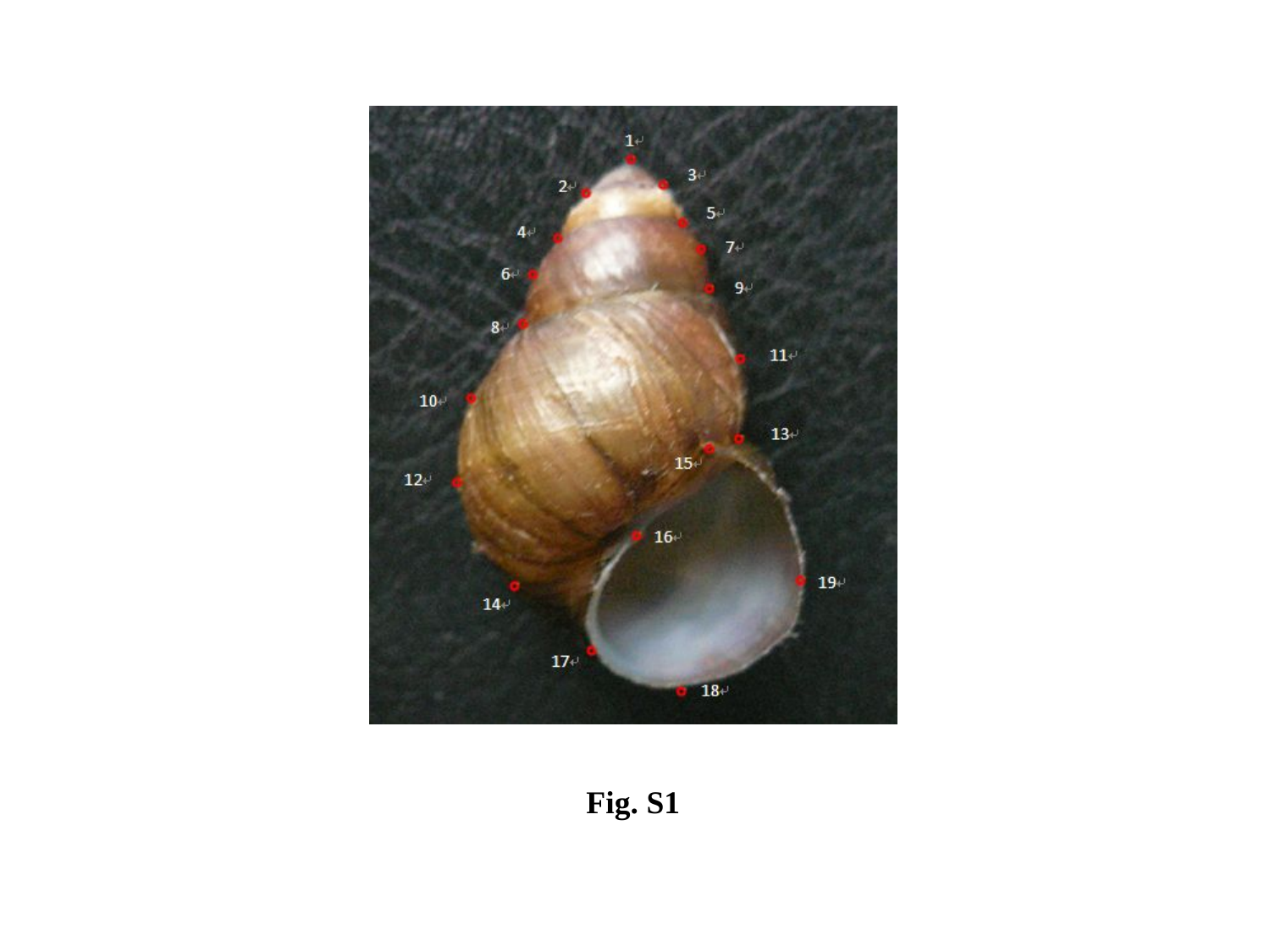

Fig. S1

Supplement: Supplementary file 7 — Figure S1. Positions of 19 landmarks superimposed on a photograph of Bellamya. (PPTX 400 kb) [file 12862_2019_1397_MOESM7_ESM.pptx]
