# Supplementary material for: Phylogeography of Bellamya (Mollusca: Gastropoda: Viviparidae) snails on different continents: contrasting patterns of diversification in China and East Africa
Source: BMC Evol Biol. 2019 Mar 21;19:82. doi: 10.1186/s12862-019-1397-0 (PMC6429760; doi:10.1186/s12862-019-1397-0)
Supplement: Supplementary file 4 — Figure S4. Median-joining haplotype network of 292 COI sequences. The size of the circles represents haplotype frequency. Each connecting line represents a single nucleotide substitution. Blue circles represent haplotype group 1 (Southeast Asia, numbers H38–138); red circles show haplotype group 2 (East Africa, numbers H1–37, except H16 and H37). In group 2, different colours represent different lake populations, Lake Malawi (red); Lake Victoria (rose red); Lake Kariba (purple); Lake Mweru (orange); Lake Tanganyika (green); Lake Bangwerulu (black); yellow represents Indian specimens (H16 and H37). For sampling details please see Fig. 4 and Additional file 5: Table S1. (PPTX 498 kb) [file 12862_2019_1397_MOESM4_ESM.pptx]

## Slide 1
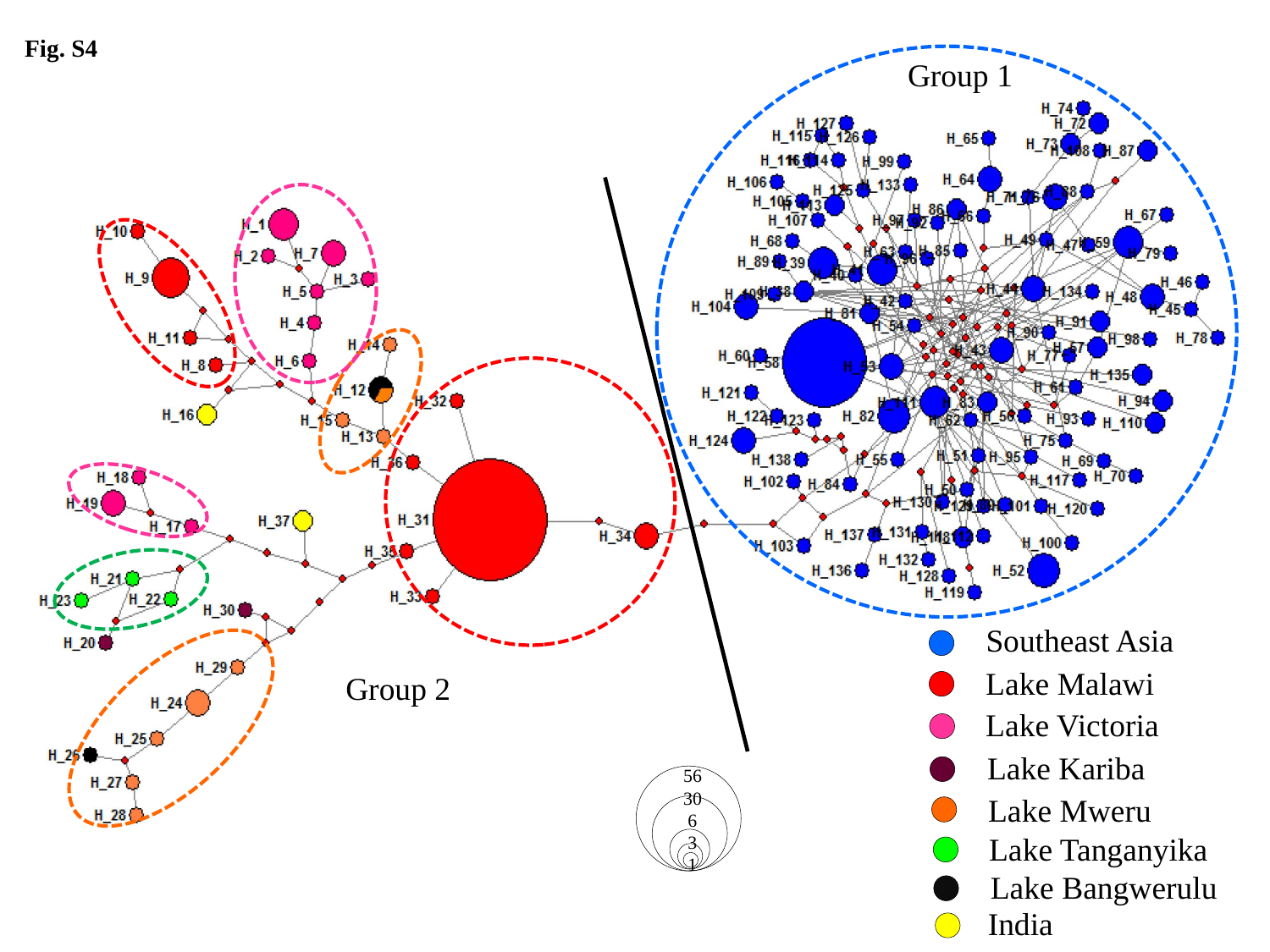

Fig. S4
Group 1
Southeast Asia
Lake Malawi
Group 2
Lake Victoria
Lake Kariba
56
30
6
3
1
Lake Mweru
Lake Tanganyika
Lake Bangwerulu
India
