# Supplementary material for: Phylogeography of Bellamya (Mollusca: Gastropoda: Viviparidae) snails on different continents: contrasting patterns of diversification in China and East Africa
Source: BMC Evol Biol. 2019 Mar 21;19:82. doi: 10.1186/s12862-019-1397-0 (PMC6429760; doi:10.1186/s12862-019-1397-0)
Supplement: Supplementary file 5 — Table S1. Taxon sampling sites, sequence IDs, and GenBank accession numbers used in this study. (DOCX 81 kb) [file 12862_2019_1397_MOESM5_ESM.docx]

**Table S1** Taxon sampling sites, sequence IDs, and GenBank accession numbers used in this study

| Unified Seq ID | *Taxon* | Country | Locality | COI | 16S | H3 | 28S |
| --- | --- | --- | --- | --- | --- | --- | --- |
| B_aeruginosa_BY1 | *B. aeruginosa* | China | Baiyangdian | KF535413 | KF535238 | KF535763 | KF535598 |
| B_aeruginosa_BY2 | *B. aeruginosa* | China | Baiyangdian | KF535414 | KF535239 | KF535764 | KF535599 |
| B_aeruginosa_BY3 | *B. aeruginosa* | China | Baiyangdian | KF535415 | KF535240 | KF535765 | KF535600 |
| B_aeruginosa_BY4 | *B. aeruginosa* | China | Baiyangdian |  | KF535241 | KF535766 | KF535601 |
| B_aeruginosa_BY5 | *B. aeruginosa* | China | Baiyangdian | KF535416 |  |  |  |
| B_aeruginosa_BY6 | *B. aeruginosa* | China | Baiyangdian | KF535417 |  |  |  |
| B_aeruginosa_CH1 | *B. aeruginosa* | China | Chaohu |  |  | KF535767 | KF535602 |
| B_aeruginosa_CH2 | *B. aeruginosa* | China | Chaohu | KF535418 | KF535242 | KF535768 | KF535603 |
| B_aeruginosa_CH3 | *B. aeruginosa* | China | Chaohu | KF535419 | KF535243 | KF535769 | KF535604 |
| B_aeruginosa_CH4 | *B. aeruginosa* | China | Chaohu | KF535420 | KF535244 | KF535770 | KF535605 |
| B_aeruginosa_CH5 | *B. aeruginosa* | China | Chaohu | KF535421 |  |  |  |
| B_aeruginosa_DC1 | *B. aeruginosa* | China | Dianchi | KF535422 | KF535245 | KF535771 | KF535606 |
| B_aeruginosa_DC2 | *B. aeruginosa* | China | Dianchi | KF535423 | KF535246 | KF535772 | KF535607 |
| B_aeruginosa_DC3 | *B. aeruginosa* | China | Dianchi | KF535424 | KF535247 | KF535773 | KF535608 |
| B_aeruginosa_DC4 | *B. aeruginosa* | China | Dianchi | KF535425 | KF535248 | KF535774 | KF535609 |
| B_aeruginosa_DC6 | *B. aeruginosa* | China | Dianchi | KF535426 |  |  |  |
| B_aeruginosa_DT4 | *B. aeruginosa* | China | Dongtinghu |  | KF535249 | KF535775 | KF535610 |
| B_aeruginosa_DT6 | *B. aeruginosa* | China | Dongtinghu | KF535427 | KF535250 | KF535776 | KF535611 |
| B_aeruginosa_DT7 | *B. aeruginosa* | China | Dongtinghu | KF535428 | KF535251 | KF535777 | KF535612 |
| B_aeruginosa_DT8 | *B. aeruginosa* | China | Dongtinghu | KF535429 | KF535252 | KF535778 | KF535613 |
| B_aeruginosa_EH1 | *B. aeruginosa* | China | Erhai | KF535431 | KF535253 | KF535779 | KF535614 |
| B_aeruginosa_EH2 | *B. aeruginosa* | China | Erhai | KF535432 |  | KF535780 | KF535615 |
| B_aeruginosa_EH3 | *B. aeruginosa* | China | Erhai | KF535433 | KF535254 | KF535781 | KF535616 |
| B_aeruginosa_EH4 | *B. aeruginosa* | China | Erhai | KF535434 | KF535255 | KF535782 | KF535617 |
| B_aeruginosa_EH5 | *B. aeruginosa* | China | Erhai | KF535435 |  |  |  |
| B_aeruginosa_EH6 | *B. aeruginosa* | China | Erhai | KF535436 |  |  |  |
| B_aeruginosa_HH1 | *B. aeruginosa* | China | Honghu | KF535437 | KF535256 | KF535783 | KF535618 |
| B_aeruginosa_HH2 | *B. aeruginosa* | China | Honghu |  | KF535257 | KF535784 | KF535619 |
| B_aeruginosa_HH3 | *B. aeruginosa* | China | Honghu | KF535438 | KF535258 | KF535785 | KF535620 |
| B_aeruginosa_HH4 | *B. aeruginosa* | China | Honghu | KF535439 | KF535259 | KF535786 | KF535621 |
| B_aeruginosa_HZ1 | *B. aeruginosa* | China | Hongzehu | KF535440 | KF535260 |  | KF535622 |
| B_aeruginosa_HZ2 | *B. aeruginosa* | China | Hongzehu | KF535441 | KF535261 | KF535787 |  |
| B_aeruginosa_HZ3 | *B. aeruginosa* | China | Hongzehu | KF535442 | KF535262 | KF535788 | KF535623 |
| B_aeruginosa_HZ4 | *B. aeruginosa* | China | Hongzehu | KF535443 | KF535263 | KF535789 | KF535624 |
| B_aeruginosa_HZ5 | *B. aeruginosa* | China | Hongzehu | KF535444 |  |  |  |
| B_aeruginosa_LZ1 | *B. aeruginosa* | China | Liangzihu | KF535445 | KF535264 | KF535790 | KF535625 |
| B_aeruginosa_LZ2 | *B. aeruginosa* | China | Liangzihu | KF535446 | KF535265 | KF535791 | KF535626 |
| B_aeruginosa_LZ3 | *B. aeruginosa* | China | Liangzihu |  |  | KF535792 | KF535627 |
| B_aeruginosa_LZ4 | *B. aeruginosa* | China | Liangzihu | KF535447 | KF535266 | KF535793 | KF535628 |
| B_aeruginosa_LZ5 | *B. aeruginosa* | China | Liangzihu | KF535448 |  |  |  |
| B_aeruginosa_LZ6 | *B. aeruginosa* | China | Liangzihu | KF535449 |  |  |  |
| B_aeruginosa_NS1 | *B. aeruginosa* | China | Nanshihu | KF535450 | KF535267 | KF535794 | KF535629 |
| B_aeruginosa_NS2 | *B. aeruginosa* | China | Nanshihu | KF535451 | KF535268 | KF535795 | KF535630 |
| B_aeruginosa_NS3 | *B. aeruginosa* | China | Nanshihu | KF535452 |  | KF535796 | KF535631 |
| B_aeruginosa_NS4 | *B. aeruginosa* | China | Nanshihu | KF535453 | KF535269 | KF535797 | KF535632 |
| B_aeruginosa_NS6 | *B. aeruginosa* | China | Nanshihu | KF535454 |  |  |  |
| B_aeruginosa_PY1 | *B. aeruginosa* | China | Poyanghu | KF535455 | KF535270 | KF535798 | KF535633 |
| B_aeruginosa_PY2 | *B. aeruginosa* | China | Poyanghu | KF535456 | KF535271 | KF535799 | KF535634 |
| B_aeruginosa_PY3 | *B. aeruginosa* | China | Poyanghu |  | KF535272 | KF535800 | KF535635 |
| B_aeruginosa_PY4 | *B. aeruginosa* | China | Poyanghu | KF535457 | KF535273 | KF535801 | KF535636 |
| B_aeruginosa_PY5 | *B. aeruginosa* | China | Poyanghu | KF535458 |  |  |  |
| B_aeruginosa_PY6 | *B. aeruginosa* | China | Poyanghu | KF535459 |  |  |  |
| B_aeruginosa_QJ1 | *B. aeruginosa* | China | Qingjiang | KF535460 | KF535274 | KF535802 | KF535637 |
| B_aeruginosa_QJ2 | *B. aeruginosa* | China | Qingjiang | KF535461 | KF535275 | KF535803 |  |
| B_aeruginosa_QJ3 | *B. aeruginosa* | China | Qingjiang | KF535462 | KF535276 | KF535804 | KF535638 |
| B_aeruginosa_QJ4 | *B. aeruginosa* | China | Qingjiang | KF535463 | KF535277 | KF535805 | KF535639 |
| B_aeruginosa_YX1 | *B. aeruginosa* | China | Yixing Taihu | KF535464 | KF535278 | KF535806 | KF535640 |
| B_aeruginosa_YX2 | *B. aeruginosa* | China | Yixing Taihu |  | KF535279 | KF535807 | KF535641 |
| B_aeruginosa_YX3 | *B. aeruginosa* | China | Yixing Taihu | KF535465 | KF535280 | KF535808 | KF535642 |
| B_aeruginosa_YX4 | *B. aeruginosa* | China | Yixing Taihu | KF535466 | KF535281 | KF535809 | KF535643 |
| B_aeruginosa_YX5 | *B. aeruginosa* | China | Yixing Taihu | KF535467 |  |  |  |
| B_aeruginosa_YX6 | *B. aeruginosa* | China | Yixing Taihu | KF535468 |  |  |  |
| B_angularis_HZ1 | *B. angularis* | China | Hongzehu | KF535546 | KF535351 | KF535875 | KF535716 |
| B_angularis_HZ2 | *B. angularis* | China | Hongzehu | KF535547 | KF535352 | KF535876 | KF535717 |
| B_angularis_HZ3 | *B. angularis* | China | Hongzehu | KF535548 | KF535353 | KF535877 | KF535718 |
| B_angularis_HZ4 | *B. angularis* | China | Hongzehu |  | KF535354 | KF535878 | KF535719 |
| B_angularis_HZ5 | *B. angularis* | China | Hongzehu | KF535549 |  |  |  |
| B_angularis_LZ1 | *B. angularis* | China | Liangzihu | KF535550 | KF535355 | KF535879 | KF535720 |
| B_angularis_LZ2 | *B. angularis* | China | Liangzihu | KF535551 | KF535356 | KF535880 | KF535721 |
| B_angularis_LZ3 | *B. angularis* | China | Liangzihu | KF535552 | KF535357 | KF535881 | KF535722 |
| B_angularis_LZ4 | *B. angularis* | China | Liangzihu | KF535553 | KF535358 | KF535882 | KF535723 |
| B_angularis_LZ6 | *B. angularis* | China | Liangzihu | KF535554 |  |  |  |
| B_angularis_NS1 | *B. angularis* | China | Nanshihu | KF535555 | KF535359 | KF535883 | KF535724 |
| B_angularis_NS2 | *B. angularis* | China | Nanshihu | KF535556 | KF535360 | KF535884 | KF535725 |
| B_angularis_NS3 | *B. angularis* | China | Nanshihu | KF535557 | KF535361 | KF535885 | KF535726 |
| B_angularis_NS4 | *B. angularis* | China | Nanshihu |  | KF535362 | KF535886 | KF535727 |
| B_angularis_NS6 | *B. angularis* | China | Nanshihu | KF535558 |  |  |  |
| B_angularis_YX1 | *B. angularis* | China | Yixing Taihu | KF535559 | KF535363 | KF535887 | KF535728 |
| B_angularis_YX2 | *B. angularis* | China | Yixing Taihu | KF535560 | KF535364 | KF535888 | KF535729 |
| B_angularis_YX3 | *B. angularis* | China | Yixing Taihu | KF535561 | KF535365 | KF535889 | KF535730 |
| B_angularis_YX4 | *B. angularis* | China | Yixing Taihu |  | KF535366 |  | KF535731 |
| B_angularis_YX5 | *B. angularis* | China | Yixing Taihu | KF535562 |  |  |  |
| B_angularis_YX6 | *B. angularis* | China | Yixing Taihu | KF535563 |  |  |  |
| B_dispiralis_CH1 | *B. dispiralis* | China | Chaohu | KF535501 | KF535310 | KF535834 | KF535671 |
| B_dispiralis_CH2 | *B. dispiralis* | China | Chaohu |  | KF535311 |  | KF535672 |
| B_dispiralis_CH3 | *B. dispiralis* | China | Chaohu | KF535502 | KF535312 | KF535835 | KF535673 |
| B_dispiralis_CH4 | *B. dispiralis* | China | Chaohu | KF535503 | KF535313 | KF535836 | KF535674 |
| B_dispiralis_CH6 | *B. dispiralis* | China | Chaohu | KF535504 |  |  |  |
| B_dispiralis_DT1 | *B. dispiralis* | China | Dongtinghu | KF535505 | KF535314 | KF535837 | KF535675 |
| B_dispiralis_DT2 | *B. dispiralis* | China | Dongtinghu | KF535506 | KF535315 | KF535838 | KF535676 |
| B_dispiralis_DT3 | *B. dispiralis* | China | Dongtinghu |  | KF535316 | KF535839 | KF535677 |
| B_dispiralis_DT4 | *B. dispiralis* | China | Dongtinghu | KF535507 | KF535317 | KF535840 | KF535678 |
| B_dispiralis_DT5 | *B. dispiralis* | China | Dongtinghu | KF535508 |  |  |  |
| B_dispiralis_PY1 | *B. dispiralis* | China | Poyanghu | KF535509 | KF535318 | KF535841 | KF535679 |
| B_dispiralis_PY2 | *B. dispiralis* | China | Poyanghu | KF535510 | KF535319 | KF535842 | KF535680 |
| B_dispiralis_PY3 | *B. dispiralis* | China | Poyanghu | KF535511 | KF535320 | KF535843 | KF535681 |
| B_dispiralis_PY4 | *B. dispiralis* | China | Poyanghu |  | KF535321 |  | KF535682 |
| B_dispiralis_PY5 | *B. dispiralis* | China | Poyanghu | KF535512 |  |  |  |
| B_dispiralis_YX1 | *B. dispiralis* | China | Yixing Taihu | KF535513 | KF535322 |  | KF535683 |
| B_dispiralis_YX2 | *B. dispiralis* | China | Yixing Taihu |  | KF535323 | KF535844 | KF535684 |
| B_dispiralis_YX3 | *B. dispiralis* | China | Yixing Taihu | KF535514 | KF535324 | KF535845 | KF535685 |
| B_dispiralis_YX4 | *B. dispiralis* | China | Yixing Taihu | KF535515 | KF535325 | KF535846 | KF535686 |
| B_dispiralis_YX5 | *B. dispiralis* | China | Yixing Taihu | KF535516 |  |  |  |
| B_dispiralis_YX6 | *B. dispiralis* | China | Yixing Taihu | KF535517 |  |  |  |
| B_lapidea_HZ1 | *B. lapidea* | China | Hongzehu | KF535542 | KF535348 | KF535872 | KF535713 |
| B_lapidea_HZ2 | *B. lapidea* | China | Hongzehu | KF535545 | KF535349 | KF535873 | KF535714 |
| B_lapidea_HZ3 | *B. lapidea* | China | Hongzehu | KF535543 | KF535350 | KF535874 | KF535715 |
| B_lapidea_HZ6 | *B. lapidea* | China | Hongzehu | KF535544 |  |  |  |
| B_lapillorum_YX1 | *B. lapillorum* | China | Yixing Taihu | KF535564 | KF535367 | KF535890 | KF535732 |
| B_lapillorum_YX2 | *B. lapillorum* | China | Yixing Taihu | KF535565 | KF535368 | KF535891 | KF535733 |
| B_lapillorum_YX3 | *B. lapillorum* | China | Yixing Taihu | KF535566 | KF535369 | KF535892 | KF535734 |
| B_lapillorum_YX4 | *B. lapillorum* | China | Yixing Taihu | KF535567 | KF535370 | KF535893 | KF535735 |
| B_lapillorum_YX5 | *B. lapillorum* | China | Yixing Taihu | KF535568 |  |  |  |
| B_lapillorum_YX6 | *B. lapillorum* | China | Yixing Taihu | KF535569 |  |  |  |
| B_purificata_BY6 | *B. purificata* | China | Baiyangdian | KF535376 |  |  |  |
| B_purificata_BY1 | *B. purificata* | China | Baiyangdian | KF535371 | KF535211 | KF535736 | KF535570 |
| B_purificata_BY2 | *B. purificata* | China | Baiyangdian | KF535372 | KF535212 | KF535737 | KF535571 |
| B_purificata_BY3 | *B. purificata* | China | Baiyangdian | KF535373 | KF535213 | KF535738 | KF535572 |
| B_purificata_BY4 | *B. purificata* | China | Baiyangdian | KF535374 | KF535214 | KF535739 | KF535573 |
| B_purificata_BY5 | *B. purificata* | China | Baiyangdian | KF535375 |  |  |  |
| B_purificata_DT1 | *B. purificata* | China | Dongtinghu | KF535377 | KF535215 | KF535740 | KF535574 |
| B_purificata_DT2 | *B. purificata* | China | Dongtinghu | KF535378 | KF535216 | KF535741 | KF535575 |
| B_purificata_DT3 | *B. purificata* | China | Dongtinghu | KF535379 | KF535217 | KF535742 | KF535576 |
| B_purificata_DT4 | *B. purificata* | China | Dongtinghu |  | KF535218 | KF535743 | KF535577 |
| B_purificata_DT5 | *B. purificata* | China | Dongtinghu | KF535380 |  |  |  |
| B_purificata_DT8 | *B. purificata* | China | Dongtinghu | KF535381 |  |  |  |
| B_purificata_DT9 | *B. purificata* | China | Dongtinghu | KF535382 |  |  |  |
| B_purificata_EH1 | *B. purificata* | China | Erhai | KF535383 | KF535219 | KF535744 | KF535578 |
| B_purificata_EH2 | *B. purificata* | China | Erhai | KF535384 | KF535220 | KF535745 | KF535579 |
| B_purificata_EH3 | *B. purificata* | China | Erhai |  |  | KF535746 |  |
| B_purificata_EH4 | *B. purificata* | China | Erhai | KF535385 | KF535221 | KF535747 | KF535580 |
| B_purificata_EH6 | *B. purificata* | China | Erhai | KF535386 |  |  |  |
| B_purificata_LZ1 | *B. purificata* | China | Liangzihu | KF535387 | KF535222 | KF535748 | KF535581 |
| B_purificata_LZ2 | *B. purificata* | China | Liangzihu | KF535388 | KF535223 | KF535749 | KF535582 |
| B_purificata_LZ3 | *B. purificata* | China | Liangzihu | KF535389 | KF535224 | KF535750 | KF535583 |
| B_purificata_LZ4 | *B. purificata* | China | Liangzihu | KF535390 | KF535225 | KF535751 | KF535584 |
| B_purificata_LZ5 | *B. purificata* | China | Liangzihu | KF535391 |  |  |  |
| B_purificata_LZ6 | *B. purificata* | China | Liangzihu | KF535392 |  |  |  |
| B_purificata_NS1 | *B. purificata* | China | Nanshihu | KF535393 | KF535226 | KF535752 | KF535585 |
| B_purificata_NS2 | *B. purificata* | China | Nanshihu |  |  | KF535753 | KF535586 |
| B_purificata_NS3 | *B. purificata* | China | Nanshihu | KF535394 | KF535227 | KF535754 | KF535587 |
| B_purificata_NS4 | *B. purificata* | China | Nanshihu | KF535395 | KF535228 | KF535755 | KF535588 |
| B_purificata_NS5 | *B. purificata* | China | Nanshihu | KF535396 |  |  |  |
| B_purificata_NS6 | *B. purificata* | China | Nanshihu | KF535397 |  |  |  |
| B_purificata_PY1 | *B. purificata* | China | Poyanghu | KF535398 | KF535229 |  | KF535589 |
| B_purificata_PY2 | *B. purificata* | China | Poyanghu | KF535399 | KF535230 | KF535756 | KF535590 |
| B_purificata_PY3 | *B. purificata* | China | Poyanghu | KF535400 | KF535231 | KF535757 | KF535591 |
| B_purificata_PY4 | *B. purificata* | China | Poyanghu | KF535401 |  |  | KF535592 |
| B_purificata_PY5 | *B. purificata* | China | Poyanghu | KF535402 |  |  |  |
| B_purificata_PY6 | *B. purificata* | China | Poyanghu | KF535403 |  |  |  |
| B_purificata_QJ1 | *B. purificata* | China | Qingjiang | KF535404 | KF535232 |  |  |
| B_purificata_QJ4 | *B. purificata* | China | Qingjiang | KF535405 | KF535233 | KF535758 | KF535593 |
| B_purificata_QJ8 | *B. purificata* | China | Qingjiang | KF535406 |  |  |  |
| B_purificata_YX1 | *B. purificata* | China | Yixing Taihu | KF535407 | KF535234 | KF535759 | KF535594 |
| B_purificata_YX2 | *B. purificata* | China | Yixing Taihu | KF535408 | KF535235 | KF535760 | KF535595 |
| B_purificata_YX3 | *B. purificata* | China | Yixing Taihu | KF535409 | KF535236 | KF535761 | KF535596 |
| B_purificata_YX4 | *B. purificata* | China | Yixing Taihu | KF535410 | KF535237 | KF535762 | KF535597 |
| B_purificata_YX5 | *B. purificata* | China | Yixing Taihu | KF535411 |  |  |  |
| B_purificata_YX6 | *B. purificata* | China | Yixing Taihu | KF535412 |  |  |  |
| B_quadrata_CH1 | *B. quadrata* | China | Chaohu | KF535469 | KF535282 | KF535810 | KF535644 |
| B_quadrata_CH2 | *B. quadrata* | China | Chaohu |  | KF535283 | KF535811 | KF535645 |
| B_quadrata_CH3 | *B. quadrata* | China | Chaohu | KF535470 | KF535284 | KF535812 | KF535646 |
| B_quadrata_CH4 | *B. quadrata* | China | Chaohu | KF535471 | KF535285 | KF535813 | KF535647 |
| B_quadrata_CH5 | *B. quadrata* | China | Chaohu | KF535472 |  |  |  |
| B_quadrata_DC1 | *B. quadrata* | China | Dianchi | KF535473 | KF535286 | KF535814 | KF535648 |
| B_quadrata_DC2 | *B. quadrata* | China | Dianchi | KF535474 | KF535287 | KF535815 | KF535649 |
| B_quadrata_DC3 | *B. quadrata* | China | Dianchi | KF535475 | KF535288 | KF535816 | KF535650 |
| B_quadrata_DC4 | *B. quadrata* | China | Dianchi |  | KF535289 |  | KF535651 |
| B_quadrata_DC5 | *B. quadrata* | China | Dianchi | KF535476 |  |  |  |
| B_quadrata_DT1 | *B. quadrata* | China | Dongtinghu | KF535477 |  |  |  |
| B_quadrata_DT2 | *B. quadrata* | China | Dongtinghu |  | KF535290 |  |  |
| B_quadrata_DT3 | *B. quadrata* | China | Dongtinghu | KF535478 |  | KF535817 | KF535652 |
| B_quadrata_DT4 | *B. quadrata* | China | Dongtinghu | KF535479 | KF535291 | KF535818 | KF535653 |
| B_quadrata_DT6 | *B. quadrata* | China | Dongtinghu | KF535480 | KF535292 |  | KF535654 |
| B_quadrata_DT7 | *B. quadrata* | China | Dongtinghu | KF535481 | KF535293 |  | KF535655 |
| B_quadrata_LZ1 | *B. quadrata* | China | Liangzihu | KF535482 | KF535294 | KF535819 | KF535656 |
| B_quadrata_LZ2 | *B. quadrata* | China | Liangzihu | KF535483 | KF535295 | KF535820 | KF535657 |
| B_quadrata_LZ3 | *B. quadrata* | China | Liangzihu | KF535484 | KF535296 | KF535821 | KF535658 |
| B_quadrata_LZ4 | *B. quadrata* | China | Liangzihu | KF535485 | KF535297 | KF535822 | KF535659 |
| B_quadrata_LZ5 | *B. quadrata* | China | Liangzihu | KF535486 |  |  |  |
| B_quadrata_LZ6 | *B. quadrata* | China | Liangzihu | KF535487 |  |  |  |
| B_quadrata_NS1 | *B. quadrata* | China | Nanshihu | KF535488 | KF535298 | KF535823 | KF535660 |
| B_quadrata_NS2 | *B. quadrata* | China | Nanshihu | KF535489 | KF535299 | KF535824 | KF535661 |
| B_quadrata_NS3 | *B. quadrata* | China | Nanshihu | KF535490 | KF535300 | KF535825 | KF535662 |
| B_quadrata_NS4 | *B. quadrata* | China | Nanshihu | KF535491 | KF535301 | KF535826 | KF535663 |
| B_quadrata_PY1 | *B. quadrata* | China | Poyanghu | KF535492 | KF535302 | KF535827 | KF535664 |
| B_quadrata_PY2 | *B. quadrata* | China | Poyanghu | KF535493 | KF535303 | KF535828 | KF535665 |
| B_quadrata_PY3 | *B. quadrata* | China | Poyanghu |  | KF535304 | KF535829 |  |
| B_quadrata_PY4 | *B. quadrata* | China | Poyanghu | KF535494 | KF535305 | KF535830 | KF535666 |
| B_quadrata_PY5 | *B. quadrata* | China | Poyanghu | KF535495 |  |  |  |
| B_quadrata_YX1 | *B. quadrata* | China | Yixing Taihu | KF535496 | KF535306 |  | KF535667 |
| B_quadrata_YX2 | *B. quadrata* | China | Yixing Taihu | KF535497 | KF535307 | KF535831 | KF535668 |
| B_quadrata_YX3 | *B. quadrata* | China | Yixing Taihu | KF535498 | KF535308 | KF535832 | KF535669 |
| B_quadrata_YX4 | *B. quadrata* | China | Yixing Taihu | KF535499 | KF535309 | KF535833 | KF535670 |
| B_quadrata_YX6 | *B. quadrata* | China | Yixing Taihu | KF535500 |  |  |  |
| B_turritus_EH1 | *B. turritus* | China | Erhai | KF535518 | KF535326 | KF535847 | KF535687 |
| B_turritus_EH2 | *B. turritus* | China | Erhai | KF535519 | KF535327 | KF535848 | KF535688 |
| B_turritus_EH3 | *B. turritus* | China | Erhai | KF535520 | KF535328 | KF535849 | KF535689 |
| B_turritus_EH4 | *B. turritus* | China | Erhai |  |  | KF535850 | KF535690 |
| B_turritus_EH5 | *B. turritus* | China | Erhai | KF535521 |  |  |  |
| B_turritus_EH6 | *B. turritus* | China | Erhai | KF535522 |  |  |  |
| V_contectus_DH01 | *V. contectus* | Denmark | Hellebaek lakes | FJ405835 | FJ405692 | FJ405737 | FJ405634 |
| B_monardi_Bo01 | *B. monardi* | Botswana | Zambezi | HQ012798 | HQ012684 |  | HQ012708 |
| B_monardi_Bo04 | *B. monardi* | Botswana | Zambezi | HQ012718 | HQ012687 |  |  |
| B_bengalensis_IS01 | *B. bengalensis* | India | Sunderbans1 | FJ405877 | FJ405724 | FJ405766 | FJ405622 |
| B_bengalensis_IS02 | *B. bengalensis* | India | Sunderbans2 | FJ405878 | FJ405735 | FJ405765 | FJ405623 |
| B_bengalensis_IS03 | *B. bengalensis* | India |  | FJ405823 |  |  |  |
| B_bengalensis_IS04 | *B. bengalensis* | India |  | FJ405822 |  |  |  |
| B_capillata_LM39 | *B. capillata* | Malawi |  | HQ012741 |  |  |  |
| B_capillata_LM02 | *B. capillata* | Malawi |  | HQ012742 |  |  |  |
| B_capillata_LM03 | *B. capillata* | Malawi |  | HQ012743 |  |  |  |
| B_capillata_LM04 | *B. capillata* | Malawi |  | HQ012744 |  |  |  |
| B_capillata_LM05 | *B. capillata* | Malawi |  | HQ012764 |  |  |  |
| B_capillata_LM06 | *B. capillata* | Malawi |  | HQ012765 |  |  |  |
| B_capillata_LM07 | *B. capillata* | Malawi |  | HQ012766 |  |  |  |
| B_capillata_LM08 | *B. capillata* | Malawi |  | HQ012767 |  |  |  |
| B_capillata_LM09 | *B. capillata* | Malawi |  | HQ012769 | HQ012703 |  |  |
| B_capillata_LM10 | *B. capillata* | Malawi |  | HQ012768 |  |  |  |
| B_capillata_LM11 | *B. capillata* | Malawi |  | HQ012773 |  |  |  |
| B_capillata_LM12 | *B. capillata* | Malawi |  | HQ012774 |  |  |  |
| B_capillata_LM13 | *B. capillata* | Malawi |  | HQ012775 |  |  |  |
| B_capillata_LM14 | *B. capillata* | Malawi |  | HQ012776 |  |  |  |
| B_capillata_LM15 | *B. capillata* | Malawi |  | HQ012777 |  |  |  |
| B_capillata_LM16 | *B. capillata* | Malawi |  | HQ012778 |  |  |  |
| B_capillata_LM17 | *B. capillata* | Malawi |  | HQ012760 |  |  |  |
| B_capillata_LM18 | *B. capillata* | Malawi |  | HQ012761 |  |  |  |
| B_capillata_LM19 | *B. capillata* | Malawi |  | HQ012762 |  |  |  |
| B_capillata_LM20 | *B. capillata* | Malawi |  | HQ012763 |  |  |  |
| B_capillata_LM21 | *B. capillata* | Malawi |  | HQ012759 |  |  |  |
| B_capillata_LM22 | *B. capillata* | Malawi |  | HQ012734 | HQ012699 |  | HQ012711 |
| B_capillata_LM23 | *B. capillata* | Malawi |  | HQ012757 |  |  |  |
| B_capillata_LM24 | *B. capillata* | Malawi |  | HQ012747 |  |  |  |
| B_capillata_LM25 | *B. capillata* | Malawi |  | HQ012738 |  |  |  |
| B_capillata_LM26 | *B. capillata* | Malawi |  | HQ012739 |  |  |  |
| B_capillata_LM27 | *B. capillata* | Malawi |  | HQ012770 |  |  |  |
| B_capillata_LM28 | *B. capillata* | Malawi |  | HQ012737 | HQ012702 |  |  |
| B_capillata_LM29 | *B. capillata* | Malawi |  | HQ012771 |  |  |  |
| B_capillata_LM30 | *B. capillata* | Malawi |  | HQ012753 |  |  |  |
| B_capillata_LM31 | *B. capillata* | Malawi |  | HQ012756 |  |  |  |
| B_capillata_LM32 | *B. capillata* | Malawi |  | HQ012754 |  |  |  |
| B_capillata_LM33 | *B. capillata* | Malawi |  | HQ012755 |  |  |  |
| B_capillata_LM34 | *B. capillata* | Malawi |  | HQ012784 |  |  |  |
| B_capillata_LM35 | *B. capillata* | Malawi |  | HQ012785 |  |  |  |
| B_capillata_LM36 | *B. capillata* | Malawi |  | HQ012786 |  |  |  |
| B_capillata_LM37 | *B. capillata* | Malawi |  | HQ012787 |  |  |  |
| B_capillata_LM38 | *B. capillata* | Malawi |  | HQ012788 |  |  |  |
| B_ecclesi_LM_01 | *B. ecclesi* | Malawi |  | HQ012733 | HQ012697 |  |  |
| B_jeffreysi_LM01 | *B. jeffreysi* | Malawi | Senga Bay | FJ405856 | FJ405690 |  | FJ405590 |
| B_jeffreysi_LM02 | *B. jeffreysi* | Malawi | Cape Mclear | FJ405873 | FJ405719 | FJ405752 | FJ405582 |
| B_jeffreysi_LM03 | *B. jeffreysi* | Malawi | Likoma Island |  |  |  | FJ405589 |
| B_jeffreysi_LM09 | *B. jeffreysi* | Malawi |  | HQ012736 | HQ012701 |  |  |
| B_jeffreysi_LM10 | *B. jeffreysi* | Malawi |  | HQ012749 |  |  |  |
| B_jeffreysi_LM11 | *B. jeffreysi* | Malawi |  | HQ012748 |  |  |  |
| B_jeffreysi_LM12 | *B. jeffreysi* | Malawi |  | HQ012750 |  |  |  |
| B_jeffreysi_LM13 | *B. jeffreysi* | Malawi |  | HQ012751 |  |  |  |
| B_jeffreysi_LM14 | *B. jeffreysi* | Malawi |  | HQ012735 | HQ012700 |  |  |
| B_robertsoni_LM01 | *B. robertsoni* | Malawi | Chirumbo Bay | FJ405871 |  | FJ405747 | FJ405581 |
| B_robertsoni_LM02 | *B. robertsoni* | Malawi |  | HQ012745 |  |  |  |
| B_robertsoni_LM03 | *B. robertsoni* | Malawi |  | HQ012746 |  |  |  |
| B_robertsoni_LM04 | *B. robertsoni* | Malawi |  | HQ012772 |  |  |  |
| B_robertsoni_LM05 | *B. robertsoni* | Malawi |  | HQ012780 |  |  |  |
| B_robertsoni_LM06 | *B. robertsoni* | Malawi |  | HQ012781 |  |  |  |
| B_robertsoni_LM07 | *B. robertsoni* | Malawi |  | HQ012779 |  |  |  |
| B_robertsoni_LM08 | *B. robertsoni* | Malawi |  | HQ012752 | HQ012698 |  |  |
| B_robertsoni_LM09 | *B. robertsoni* | Malawi |  | HQ012740 |  |  |  |
| B_robertsoni_LM10 | *B. robertsoni* | Malawi |  | HQ012789 |  |  |  |
| B_robertsoni_LM11 | *B. robertsoni* | Malawi |  | HQ012790 |  |  |  |
| B_robertsoni_LM12 | *B. robertsoni* | Malawi |  | HQ012791 |  |  |  |
| B_robertsoni_LM13 | *B. robertsoni* | Malawi |  | HQ012792 |  |  |  |
| B_robertsoni_LM14 | *B. robertsoni* | Malawi |  | HQ012793 |  |  |  |
| B_robertsoni_LM15 | *B. robertsoni* | Malawi |  | HQ012794 |  |  |  |
| B_robertsoni_LM16 | *B. robertsoni* | Malawi |  | HQ012795 |  |  |  |
| B_robertsoni_LM17 | *B. robertsoni* | Malawi |  | HQ012796 |  |  |  |
| B_robertsoni_LM18 | *B. robertsoni* | Malawi |  | HQ012797 | HQ012704 |  |  |
| B_robertsoni_LM19 | *B. robertsoni* | Malawi |  | HQ012758 |  |  |  |
| B_monardi_Bo03 | *B. monardi* | Namibia | Okavango Delta | HQ012800 | HQ012686 |  | HQ012709 |
| V_ater_SZ01 | *Viviparus ater* | Switzerland | Lake Zurich,Goldbach | FJ405882 | FJ405734 | FJ405774 | FJ405630 |
| B_capillata_LM01 | *B. capillata* | Tanzania | Nsesi1 | FJ405858 | FJ405717 |  | FJ405588 |
| B_costulata_LV12 | *B. costulata* | Tanzania | Lake Victoria | HQ012712 | HQ012680 |  |  |
| B_jeffreysi_LM04 | *B. jeffreysi* | Tanzania | Mwaya | FJ405865 |  | FJ405760 | FJ405586 |
| B_jeffreysi_LM05 | *B. jeffreysi* | Tanzania | River Songwe | FJ405857 | FJ405702 | FJ405763 | FJ405583 |
| B_jeffreysi_LM06 | *B. jeffreysi* | Tanzania | Nsesi2 | FJ405864 |  |  | FJ405587 |
| B_jeffreysi_LM07 | *B. jeffreysi* | Tanzania | Njisi | FJ405859 | FJ405700 | FJ405762 | FJ405584 |
| B_jeffreysi_LM08 | *B. jeffreysi* | Tanzania | Lufilyo | FJ405860 | FJ405701 | FJ405761 | FJ405585 |
| N_tanganyicense_LT02 | *N. tanganyicense* | Tanzania | Lake Tanganyika | HQ012716 | HQ012683 |  | HQ012706 |
| N_tanganyicense_LT03 | *N. tanganyicense* | Tanzania | Lake Tanganyika | HQ012717 |  |  | HQ012707 |
| B_rubicunda_LA01 | *B. rubicunda* | Uganda | Butiaba | FJ405836 | FJ405715 | FJ405750 |  |
| B_rubicunda_LA02 | *B. rubicunda* | Uganda | Rwangala | EU274556 |  | EU274502 | EU274517 |
| B_rubicunda_LA03 | *B. rubicunda* | Uganda | Bugoigo | FJ405861 | FJ405716 |  | FJ405619 |
| B_rubicunda_LA04 | *B. rubicunda* | Uganda | Lake Albert | HQ012714 | HQ012682 |  |  |
| B_rubicunda_LA05 | *B. rubicunda* | Uganda | Lake Albert | HQ012715 | HQ012681 |  | HQ012705 |
| B_rubicunda_LA06 | *B. rubicunda* | Uganda | Lake Albert | HQ012713 |  |  |  |
| B_trochlearis_LV01 | *B. trochlearis* | Uganda | Bukakata | FJ405837 | FJ405691 | FJ405741 | FJ405608 |
| B_trochlearis_LV02 | *B. trochlearis* | Uganda | Lambu | FJ405846 | FJ405704 | FJ405748 | FJ405612 |
| B_trochlearis_LV03 | *B. trochlearis* | Uganda | Kivindi | FJ405870 |  |  | FJ405602 |
| B_trochlearis_LV04 | *B. trochlearis* | Uganda | Ngombe | FJ405855 | FJ405698 | FJ405759 | FJ405603 |
| B_trochlearis_LV05 | *B. trochlearis* | Uganda | Lugala | FJ405847 | FJ405714 |  | FJ405613 |
| B_unicolor_Nl | *B. unicolor elatior* | Uganda | Kumucanga,Victoria Nile | FJ405851 | FJ405708 | FJ405742 | FJ405617 |
| B_trochlearis_LV06 | *B. trochlearis* | Uganda | Lake Victoria | FJ405847 | FJ405714 |  | FJ405613 |
| B_unicolor_LKy01 | *B. unicolor* | Uganda | Lyingo | FJ405863 | FJ405713 |  | FJ405607 |
| B_unicolor_LV01 | *B. unicolor elatior* | Uganda | Jinja Fishery1 | FJ405848 | FJ405705 |  | FJ405614 |
| B_unicolor_LV02 | *B. unicolor elatior* | Uganda | Jinja Fishery2 | FJ405853 | FJ405697 | FJ405743 | FJ405615 |
| B_capillata_LBa01 | *B. capillata* | Zambia | Mwense Village, Samfya | FJ405874 | FJ405693 | FJ405754 | FJ405594 |
| B_capillata_LBa02 | *B. capillata kalungwisi* | Zambia | Kalungwishi River | FJ405841 | FJ405696 | FJ405744 | FJ405597 |
| B_crawshayi_LMw01 | *B. crawshayi* | Zambia | Mkolo1 | FJ405844 | FJ405695 | FJ405746 | FJ405596 |
| B_crawshayi_LMw02 | *B. crawshayi* | Zambia | Mkolo2 | FJ405867 | FJ405710 |  | FJ405591 |
| B_crawshayi_LMw03 | *B. crawshayi* | Zambia | Lake Mweru | FJ405844 | FJ405695 |  | FJ405596 |
| B_crawshayi_LMw04 | *B. crawshayi* | Zambia | Lake Mweru | FJ405867 | FJ405710 |  | FJ405591 |
| B_monardi_Bo02 | *B. monardi* | Zambia | Zambezi | HQ012799 | HQ012685 |  |  |
| B_mweruensis_LMw01 | *B. mweruensis* | Zambia | WT-guesthouse |  |  |  | FJ405593 |
| B_mweruensis_LMw02 | *B. mweruensis* | Zambia | Shimbofuma | FJ405842 |  |  | FJ405592 |
| B_mweruensis_LMw03 | *B. mweruensis* | Zambia | Lake Mweru | FJ405842 |  |  | FJ405592 |
| B_pagodiformis_LMw01 | *B. pagodiformis* | Zambia | Isokwe Island | FJ405840 | FJ405694 |  | FJ405595 |
| B_pagodiformis_LMw02 | *B. pagodiformis* | Zambia | Lake Mweru | HQ012719 |  |  |  |
| B_pagodiformis_LMw03 | *B. pagodiformis* | Zambia | Lake Mweru | HQ012720 | HQ012688 |  |  |
| B_pagodiformis_LMw04 | *B. pagodiformis* | Zambia | Lake Mweru | HQ012721 | HQ012689 |  |  |
| N_tanganyicense_LT01 | *Neothauma tanganyicense* | Zambia | Mpulungu | FJ405843 | FJ405709 | FJ405739 | FJ405598 |
| N_tanganyicense_LT04 | *N. tanganyicense* | Zambia | Lake Tanganyika | FJ405843 | FJ405709 |  | FJ405598 |
| B_capillata_LKa01 | *B. capillata* | Zimbabwe | Chawara | FJ405838 | FJ405689 | FJ405745 | FJ405620 |
